# Mechanical Properties of a 3D-Printed Wall Segment Made with an Earthen Mixture

**DOI:** 10.3390/ma15020438

**Published:** 2022-01-07

**Authors:** Elena Ferretti, Massimo Moretti, Alberto Chiusoli, Lapo Naldoni, Francesco De Fabritiis, Massimo Visonà

**Affiliations:** 1 Department of Civil, Environmental and Materials Engineering—DICAM, Alma Mater Studiorum Università di Bologna, Viale del Risorgimento 2, 40136 Bologna, Italy; 2 WASP s.r.l., Via Castelletto 104/106, 48024 Massa Lombarda, Italy; massimo@3dwasp.com (M.M.); alberto@3dwasp.com (A.C.); lapo@3dwasp.com (L.N.); francesco@3dwasp.com (F.D.F.); massimo.visona@3dwasp.com (M.V.)

**Keywords:** 3D printing, earthen buildings, biocomposites, load-bearing walls, orthotropic material, crack propagation, Poisson’s ratio, volumetric strain, mesoscale behavior, microscale behavior

## Abstract

This study provides a contribution to the research field of 3D-printed earthen buildings, focusing, for the first time, on the load-bearing capacity of these structures. The study involves the entire production and testing process of the earthen elements, from the design, to the preparation of the mixture and the 3D printing, up to the uniaxial compression test on a wall segment. The results indicate that 3D-printed earthen elements have a compressive strength of 2.32 MPa, comparable to that of rammed earth structures. The experimental data also made it possible to draw conclusions on the action of the infill, which seems to have the function of stopping the propagation of cracks. This has a positive effect on the overall behavior of 3D-printed earthen elements, since it avoids the onset of dilative behavior in the final stages of the load test and maintains ultimate load values higher than 50% of the maximum load.

## 1. Introduction

Earthen construction (i.e., the construction of structural units manufactured from soil) is one of the oldest and most widespread vernacular building techniques. Over the centuries, it has given rise to three main traditional construction techniques: adobe (or compressed earth blocks), rammed earth and cob.

The advent of industrialization has progressively supplanted the earthen construction techniques, which are considered expressions of building in poverty conditions, overtaken by progress and well-being. Recently, however, earthen construction has regained a lot of attention in the construction sector, due to its low environmental impact and its recyclability [1,2,3,4]. In fact, earthen structures need a fraction of the energy required to manufacture and process an equivalent amount of fired bricks or concrete (low embodied energy) [5,6,7], require lower carbon emissions for construction (low carbon footprint) [8] and consume less operational energy than conventional structures (high energy efficiency) [9]. Finally, earthen materials also need less energy to recycle them [10]. Nevertheless, the cost of labor, the time it takes for the material to harden and a slower production rate than in the concrete industry result in high finished-product costs, which have limited the development of earthen construction in modern times [11]. The problem of slowness in production is particularly problematic to solve, since fast production usually does not allow the achievement of sufficient strength in the dry state. Therefore, the primary concern in setting up a mix design for an earthen mixture is to provide a viable solution to both the production rate problem and the dry-state strength problem.

Among the recently introduced unconventional methods to improve construction speed with earth [12,13,14,15], digital-based construction methods, such as 3D printing, are particularly promising. As with extrusion-based additive manufacturing methods for concrete [16,17], the quality of the 3D printing process for earthen materials is the result of a trade-off between two competing requirements: material deposition speed and construction speed. This means that the setting of the material must begin as soon as possible, so that the deposited material can withstand the increasing load induced by the subsequently deposited layers. Since the material must also have high strengths in the hardened (dry) state [18,19], the 3D printing process of earthen materials should integrate the research line on soil stabilization. In fact, this line of research—originally created for traditional earthen structures [20]—boasts many years of study on techniques suitable for improving the mechanical properties of earthen structures in the hardened state. This has led researchers, for example, to investigate the role of different stabilizers in the preparation of Compressed Stabilized Earth Blocks (CSEBs) [21,22,23] and to identify the optimum use of stabilizers [24,25,26,27] in order to meet the criteria of the local building code [28].

From the point of view of the evolution of the soil stabilization techniques, soil stabilization first made use of stabilizers of natural origin—such as the addition of vegetable fibers [29,30] and biomaterial in the form of egg whites or proteins [31,32,33]—and, subsequently, of industrial stabilizers, such as lime [34] and Portland cement [35]. However, these remedies do not have the same impact on the environment and are not all equally sustainable [36], an aspect that has become increasingly important with the recent development of sensitivity to environmental issues. Particularly detrimental to sustainability is the use of cement, as it leads to an increase in the embodied energy, carbon footprint and operational energy and reduces the potential for recyclability [10,37,38,39]. This has led to a renewed interest in biostabilization techniques, such as the use of microbially induced calcite precipitation (MICP) [40,41,42], different types of biopolymers (for example, xanthan gum [43,44,45], gellan gum [46], agar gum [46], guar gum [47], modified starch [47] and carrageenan [48]) and biopolymer additives [49], in both the construction [50] and geotechnical [51,52,53,54] sectors. Most of these techniques derive from the knowledge gained in the research on concrete. Just to give an example, in fact, the incorporation of bacteria in rice husk ash concrete increases the strength of concrete due to the precipitation of calcium carbonate, which occurs at any age of the concrete [55,56].

One of the first companies to deal with the 3D printing of earthen materials was the Italian WASP (acronym for the World’s Advanced Saving Project), which also developed a particular type of biocomposite for the stabilization of earthen mixtures [57]. WASP has also always been committed to the development of systems that allow the use of functional, end-use materials such as ceramics, porcelain, clay and other advanced ceramics, in order to promote digital handicraft and self-production.

The WASP biocomposite is a combination of rice husk (RH) and lime. It exploits the high silica content of the RH and the time-delayed biocementation effect of the aerial lime, due to the carbonation of the lime [58]. Since the carbonation continues indefinitely over time, the products of the carbonation improve the mechanical properties of the earthen mixtures more and more over time [58], also protecting the vegetable fiber. In fact, the combination of a vegetable fiber with lime (a mineral) and water allows the vegetable fiber to mineralize and become resistant to bacteria, molds, insects, rodents and fire.

Since 2017, WASP has undertaken two different lines of research on earthen materials: the 3D printing of infill walls using natural mixtures of local origin without any type of chemical stabilization (Figure S1) and the 3D printing of load-bearing elements by additivating the mixtures with the minimum quantity of hydraulic lime-based stabilizers (Figure S2) [59]. The second line of research aims to create a new circular building model, entirely made with reusable and recyclable materials. The present work is part of this second line of research, which, of the two, is the richest in innovative aspects. Indeed, while it is quite common to find experimental results on earthen construction materials with (even unusual) organic compounds as stabilizers [60,61], and the 3D printing of earthen materials is becoming an increasingly interesting research topic [11,62,63], the mechanical characterization of 3D-printed earthen elements for load-bearing use is still largely lacking in the scientific literature. To the authors’ knowledge, this is actually the first scientific work dealing with the load-bearing capacities of 3D-printed earthen elements with bio-stabilized soil.

Since one of the major problems in regard to load-bearing earthen walls is how to increase the number of floors of the earthen building (the load-bearing prototypes made so far are single-story), it is essential to investigate the failure mechanisms of earthen walls subjected to vertical loads. Of particular interest is understanding whether the individual extruded layers collaborate as a single body to withstand external loads. Another concern relates to the behavior of the outer shells at the time of the failure. Depending on the degree of cohesion with the internal infill, in fact, the outer shells may or may not undergo a wall overturning, moving by rigid motion independently of the rest of the wall. The experimental test presented in this work answers precisely these questions, never investigated until now.

One might regard this paper as the completion of a previous work on the study of the mechanical properties over time of biostabilized earthen mixtures [58], which is the equivalent for earthen materials of the description over time of the evolution of the cementitious materials [64,65,66,67], aimed at the rapid strength development in the material for 3D printing. The idea behind the experimental campaign discussed in [58] was to increase the cementing capacity of the RH–lime biocomposite by triturating the RH, which increases the contact surface between the RH and the earthen matrix. This favors the formation of Si–O–Si bonds and improves the mechanical properties of the earthen mixture. The results of [58] showed that the stiffness values obtained with the shredded RH were actually higher than the stiffness values obtained with the nonshredded RH (all other conditions being equal, including the quantity of RH). Since this was precisely the expected result that motivated the experimentation of [58], the RH used in the wall segment of this paper (printed on the same day as the casting of the [58] specimens) is shredded RH.

## 2. Materials and Methods

To investigate the ability of 3D-printed earthen walls to withstand the vertical loads, we tested an earthen wall segment with all the characteristic thicknesses in 1:1 scale. We used, in fact, the same cross-sectional dimensions of the case studies in 1:1 scale made by WASP in the past (Figures S1 and S2).

According to the Highway Research Board (HRB) [68]/UNI EN ISO 14688-1:2018 [69] soil classification, the soil collected on site for the preparation of the mixture was class A-4. This soil class has a wide distribution in Italy, with a greater presence in the Piedmont Po Plain of north-western Italy, in correspondence with large terraced alluvial fans, perched in the plains, connected with the middle–upper Pleistocene rivers of the Upper Piedmont [70]. The biostabilization of the A-4 soil took place using the RH–lime biocomposite, according to the principles described in [58]. Since the subject of this work is the load-bearing capacity of 3D-printed earthen elements, for everything concerning the mechanical effect of the vegetable fiber on the properties of the hardened earthen mixture, we refer the reader to what we have already written in [58].

The test method chosen was the uniaxial compression test, conducted in the displacement control mode at the LiSG laboratory of the University of Bologna.

### 2.1. Mix Design and 3D-Printing Process

The mix design of the earthen wall segment was one of the two used for the cubic specimens of [58], which shows the results of an experimental campaign on earthen mixtures with RH in two different grain sizes: unaltered RH for the TQ mix and shredded RH for the LT mix. The mix chosen for the wall segment of this paper was the LT mix (Table 1), with maximum size of the shredded RH equal to 2 mm. The specifications of the other components of the LT mix are listed in Table S1 of the Supplementary Materials, to avoid repeating what was already extensively described in [58].

The preparation of the mixture and the 3D printing of the specimen took place at the WASP headquarters (Massa Lombarda, Italy).

**Figure 1:**
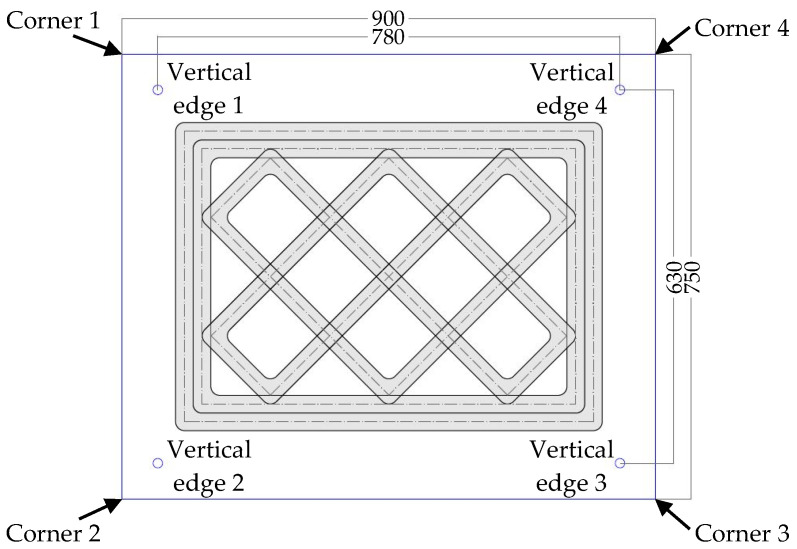
Dimensions of the specimen cross-section and of the 1.5 cm thick load-distribution plate.

The 3D-printed specimen of the experimental test was a parallelepiped with design dimensions of 730 mm × 530 mm × 504 mm (length × depth × height). It consisted of 42 extruded layers 12 mm high. The print geometry included two perimeter shells to confine the infill stratigraphy (Figure 1), which was consistent with the usual way of designing WASP 3D-printed walls in case studies since 2017. This geometric configuration with outer shells and infill was similar to that adopted in the contour-crafting technique for concrete [71,72].

The infill was a regular mesh grid, characterized by double symmetry and orthogonality of the lines. Ten equidistant straight sections 35 mm thick made up the infill, 5 with an inclination of +45° and 5 with an inclination of −45° (Figure 1). The distance between two successive intersections of the straight sections was approximately 150 mm.

To ensure a good grasp between infill and perimeter shells, the infill extended into the external cladding for 1/4 of its total thickness (Figure 1), which was equal to 68 mm (the two perimeter shells had a thickness of 34 mm each). The partial interpenetration between infill and perimeter shells resulted in 10 vertical ribs along the outer perimeter of the specimen.

Considering the curvature of the layers at the corners of the specimen, the gross area of the cross-section, $A_{g}$, was 374,400 mm^2^, while the net area (excluding voids), $A_{n}$, was 228,900 mm^2^:

(1) $$A_{g}=374,400\;{\mathrm{mm}}^{2},$$

(2) $$A_{n}=228,900{\;\mathrm{mm}}^{2},$$

(3) $$\frac{A_{n}}{A_{g}}=0.611.$$

The filling ratio of about 61% in Equation (3) is consistent with the most widely accepted standards for semisolid fired bricks and blocks for load-bearing walls [73] (there are still no standards for the filling ratio for semisolid earthen load-bearing elements). According to ASTM C 652-21 [74], for example, a hollow fired brick has a minimum filling ratio of 40% and a maximum filling ratio of 75% (coring or void area greater than 25%, with a maximum of 60%). Characteristics such as redundancy, directionality and the full/empty ratio are actually crucial to guarantee the load-bearing capacity of the 3D-printed walls. As these parameters change, the performance of 3D-printed monomaterial masonry varies, which allows the professional to design the walls for 3D printing in such a way as to achieve a wide range of functionality. Therefore, the performative characterization of the 3D-printed walls as these parameters vary is a very interesting topic for the continuation of this line of research.

**Figure 2:**
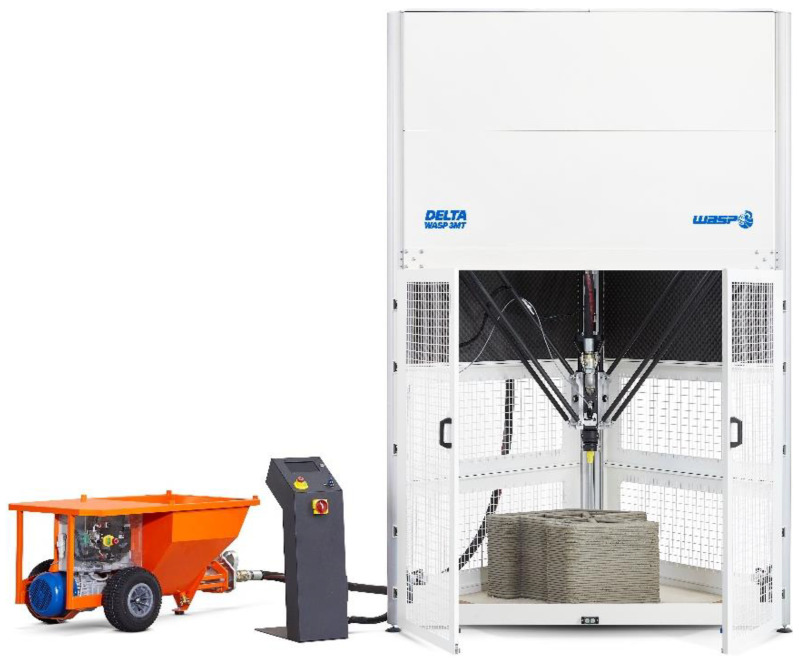
The Delta WASP 3MT Industrial 4.0 LDM (https://www.3dwasp.com/en/concrete-3d-printer-delta-wasp-3mt-concrete, accessed on 20 December 2021).

The printing session (March 2020) took place with the Delta WASP 3MT Industrial 4.0 LDM printer model (Figure 2), created by WASP (Massa Lombarda, Italy) to support research centers and laboratories in the experimentation and prototyping of components on an architectural scale. The 3D printer makes use of the Liquid Deposition Modeling (LDM) technology, which is the technology that WASP uses for its extruder for ceramic materials. Developed to achieve a level of kinematic precision very close to that of plastic polymer extruders, the LDM technology combines a screw extruder and a pressure sensor that precisely controls the flow of material and uses retraction to stop deposition. The extrusion takes place thanks to a helical screw that dispenses the right amount of material, following the instructions deriving from the G-code, moment by moment.

**Figure 3:**
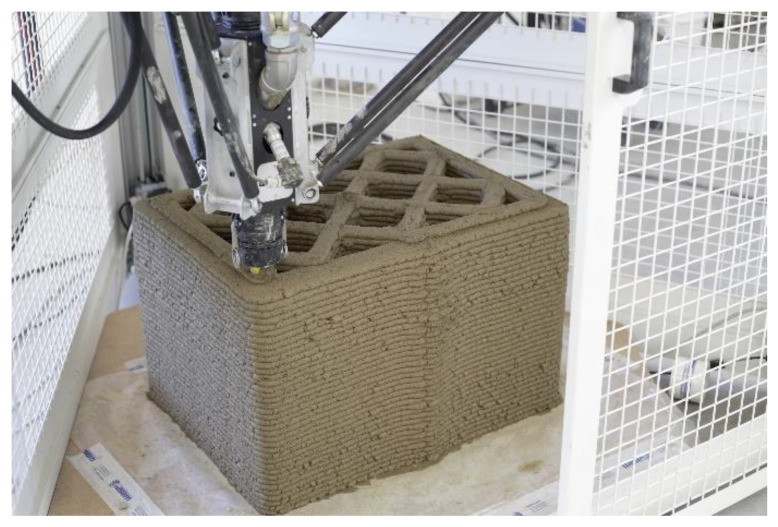
The 3D printing of the specimen object of this paper.

The Delta WASP 3D printer uses the “continuous feeding system” (Figure 2), which makes the pumping and feeding of the extruder continuous in a responsive way, thanks to a real-time reading system of the operating pressure at the inlet of the hose on the extruder. To obtain a good homogeneity and workability, the feeding of the 3D printer came directly from the muller that kneaded the mixture. As regards the specimen object of this paper, the extrusion setup was set with a nozzle of D 30 mm (Figure 3).

To allow comparison between the strength of the 3D-printed wall segment and the average strength of the first set of cubes of [58] (Section 3.4), 3D printing of the wall segment and casting of the cubes took place on the same day and all the specimens underwent a uniaxial compression test after the same curing period (90 days).

### 2.2. Feasibility Assessments

The carbonation of the aerial lime in the RH–lime biocomposite determines a production of calcium carbonate that continues indefinitely over time. This deposition mechanism is somewhat similar to the delayed precipitation of calcium carbonate that occurs due to the metabolic action of bacteria (in the MICP). These are, therefore, very similar stabilization mechanisms but, at the same time, of different implementation impact. In fact, while the MICP treatment consists of stimulating the growth of indigenous bacteria in situ (biostimulation) or increasing the ureolytic bacterial culture (bioaugmentation) [75,76], the RH–lime biocomposite is nothing more than a simple mixture of rice husk and aerial lime. Furthermore, carbonation strongly depends on the long-term stability of bacterial urease in the MICP [75,77], while it occurs spontaneously and without the need for special precautions in the RH–lime biocomposite.

The RH–lime biocomposite is also advantageous from an economic point of view. In fact, it combines lime (not particularly expensive) with RH, which is a waste product (more precisely, a waste product from rice processing). It is worth noting that there is a large availability of RH in the world, as rice cultivation produces a large volume of RH each year (20% to 25% by weight of the rice crop). Since this volume is only used to a small extent, as a source of renewable energy, RH poses environmental problems related to its disposal. The two most frequently used disposal methods, in fact, are both harmful to the environment: the first method consists of landfilling for organic waste, with the potential pollution of the soil and aquifers; the second method consists of open combustion, with the release of carbon dioxide into the atmosphere and potential contribution to global warming. Its use in the RH–lime biocomposite for construction purposes is, therefore, also a viable way to reduce the environmental impact of RH. Based on WASP’s experimental data, one hectare of cultivated paddy field is able to produce 100 square meters of built area.

**Figure 4:**
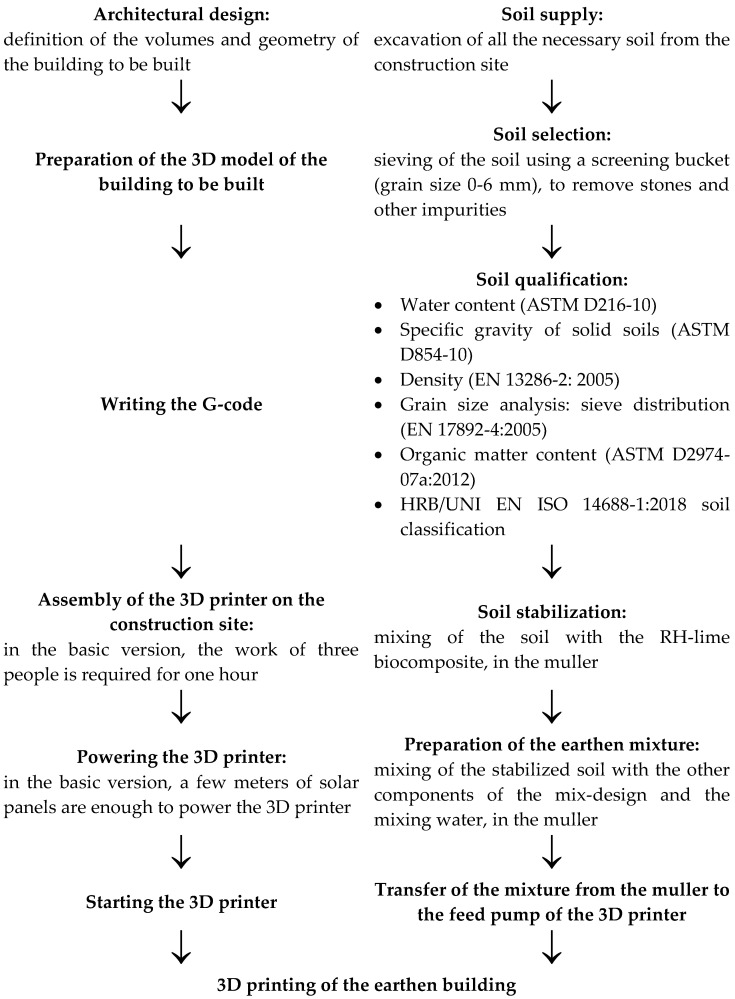
Flowchart of the 3D-printing process for earthen construction.

The construction of WASP’s two full-scale housing prototypes—Gaia (Figure S1) and Tecla (Figure S2)—is proof of the feasibility of the entire process, from the architectural design and soil supply to the 3D printing of entire housing modules (Figure 4). It is worth noting that Figure 4 shows two different mixing phases in the muller, the first aimed at stabilizing the soil and the second aimed at kneading the mixture. In reality, until now, WSAP has made use of only one mixing phase, which consists of simultaneously inserting all the components of the mix design into the muller, together with the mixing water. The opportunity to carry out the mixing at two different moments is one of the results that emerged from [58], as this seemed to favor the biocementing action of the biocomposite.

In its simplest version (Gaia), the WASP housing module requires only 100 h of printing, which allows the quick resolution of housing emergencies that may arise as a result, for example, of natural disasters.

In the WASP spirit of promoting zero-kilometer home construction, the soil used for 3D printing comes entirely from the construction site (Figure 4), which keeps construction costs very low. In fact, in addition to not incurring costs for the supply of the soil (the main component of the earthen mixture, as shown in Table 1), 3D printing with soil taken from the construction site eliminates the costs of the disposal of the excavated soil, which constitutes a large part of the expenses in a traditional construction. Furthermore, compared to traditional earthen constructions, 3D printing produces thicker walls using less soil, thanks to the honeycomb structure of the infill. This allows us to provide the walls with high moments of inertia to withstand horizontal loads, with a lower labor cost than massive traditional earthen structures.

Obviously, since Gaia and Tecla are prototypes, it is not yet possible to draw definitive conclusions on construction costs from a large-scale production perspective. In fact, the cost of the 3D-printing activity consists of various items, the incidence of which varies from case to case, based on the production setup: (i) modeling/consulting, (ii) starting the 3D printer, (iii) material and (iv) labor and fixed costs.

### 2.3. Geometric Characteristics of the Specimen in the Fresh and Hardened States

**Figure 5:**
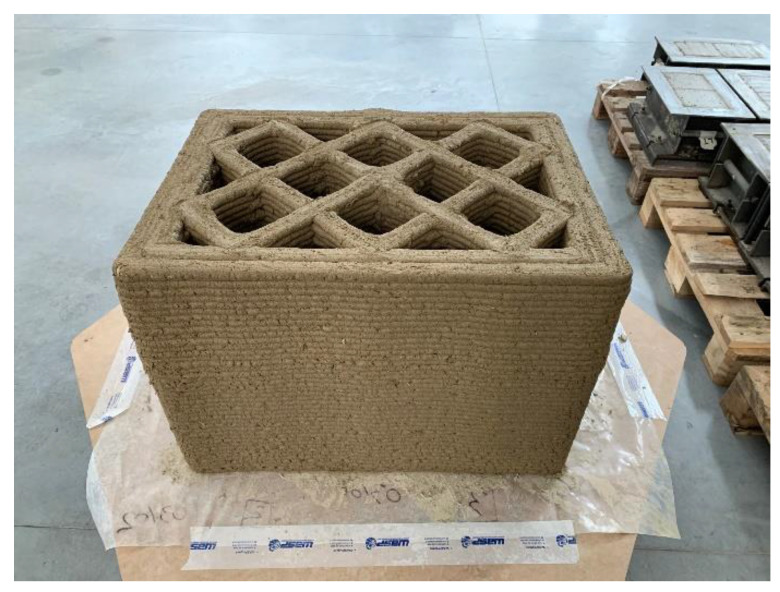
3D-printed specimen in the fresh state.

**Figure 6:**
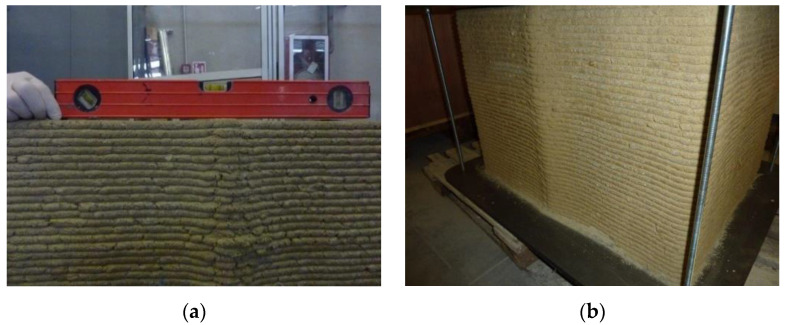
3D-printed specimen in the hardened state: (**a**) excess material on the upper face; (**b**) excess material on the rear side (long side face opposite the operator).

The fresh specimen showed ripples on the side surfaces, with a periodic pattern equal to the distance between the vertical ribs (Figure 5). Furthermore, the crossing between successive layers generated an overabundance of material that accentuated the central rib of one of the two long side faces. The enlarged rib is visible, in part, in Figure 5 (on the rear edge of the upper face) and, in more detail, in Figure 6a,b. The excess material also caused the deformation of the upper face, in correspondence with the enlarged rib (this deformation is visible in Figure 6a, which is a photo taken of the hardened state).

The shrinkage suffered by the specimen during the hardening phase did not accentuate the undulation of the lateral and upper surfaces, as the shrinkage caused the same percentage variation of the lengths along all directions. Therefore, the shrinkage caused a homothetic variation in the shape of the specimen.

The extent of shrinkage is highly dependent on the mix design, which can vary within quite wide limits in earthen mixtures, for both traditional and additive manufacturing. Thus, the percentage change in lengths caused by shrinkage is highly variable in earthen mixtures for 3D printing. This has made it impossible until now to define a standard for shrinkage in extrusion earthen mixtures.

The average value reached by the linear dimensions of the wall segment in the hardened state was approximately 97.24% of the corresponding value in the fresh state, with an average (negative) percentage variation of 2.76%. Due to the dependence of shrinkage on the mix design, this value is only comparable with the data of specimens made with the same mixture, that is, the LT mix. It is therefore comparable with the (negative) percentage variation of 2.44% undergone by the cubic specimens of [58], made with the same mixture, on the same day and in the same thermohygrometric conditions of the wall segment. Since the casting of the cubic specimens of [58] took place by hand compaction in the formworks, the good agreement between the two shrinkage values implies that the extrusion process did not significantly alter the shrinkage behavior of the earthen mixtures. Table 2 gives the main dimensions of the specimen in the hardened state.

The percentage variation of the areas is equal to the square of the percentage variation of the linear dimensions. The estimated value of the nominal area after hardening was therefore equal to:

(4) $$A_{extr}=0.9724^{2}A_{n}=216,439.09\;{\mathrm{mm}}^{2},$$

where $A_{n}$ is the nominal area in the fresh state, given by Equation (2).

**Figure 7:**
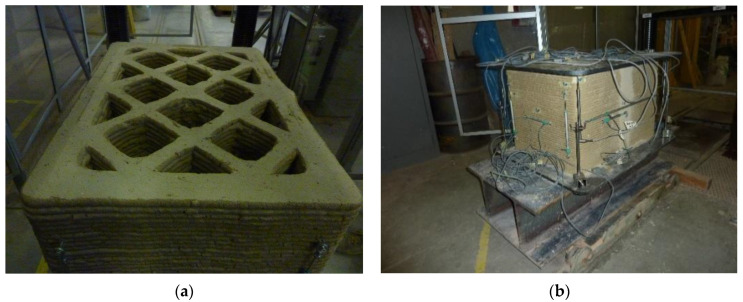
The specimen after hand sanding: (**a**) on the upper face; (**b**) on the long side face facing the operator.

To ensure a uniform distribution of the load on the upper face, it was necessary to remove the excess material on the upper face (Figure 7a) by hand sanding. This allowed the upper metal plate for load transfer (whose dimensions are given in Figure 1) to adhere perfectly to the upper face of the specimen (Figure 7b). To allow housing the instruments on the long side faces, it was also necessary to remove the excess material of the enlarged rib (Figure 7b), again by sanding.

### 2.4. Instrumentation for the Acquisition of the Displacements

The acquisition of the displacements was carried out by means of Linear Variable Differential Transformers (LVDTs) produced by Gefran SPA (Brescia, Italy), using a total of 15 instruments. To be precise, 7 were the LVDTs used for the acquisition of the horizontal displacements and 8 were those used for the vertical displacements. Furthermore, among the 7 LVDTs used for the acquisition of the horizontal displacements, 5 acquired the displacements between 2 successive vertical ribs, and the remaining 2 acquired the displacements along the entire length of the long side faces. Finally, 4 of the 8 LVDTs used for the acquisition of the vertical displacements measured the displacements along the 4 vertical edges of the specimen, and the remaining 4 measured the displacements between opposite corners of the 2 metal plates, placed one below and one above the specimen.

**Figure 8:**
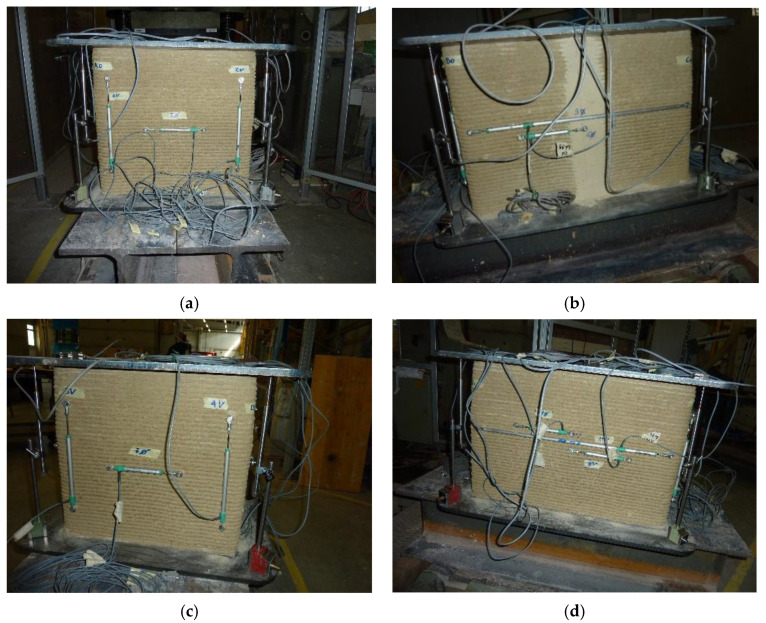
Side views of the LVDTs, listed from left to right: (**a**) LVDT VA, LVDT V1, LVDT H5, LVDT V2, LVDT VB on the short side face to the left of the operator; (**b**) LVDT VB, LVDT H9, LVDT H6, LVDT VC on the long side face in front of the operator; (**c**) LVDT VC, LVDT V3, LVDT H7, LVDT V4, LVDT VD on the short side face to the right of the operator; (**d**) LVDT VD, LVDT H10, LVDT H11, LVDT H8, LVDT VA on the long side face on the opposite side of the operator.

Table 3 provides the labeling of the 15 LVDTs and summarizes their positioning (according to the numbering of edges and corners given in Figure 1). Figure 8 shows the 15 LVDTs after their placement.

## 3. Experimental Results

The experimental results confirmed what was already concluded from the uniaxial compression tests on the cubic specimens made with the same mixture [58]. That is, although the load/displacement curve was similar to that typical for a brittle material, the overall behavior of the specimen presented peculiarities attributable to the rheology of the soils.

### 3.1. Behavior of the Vertical Strains

**Figure 9:**
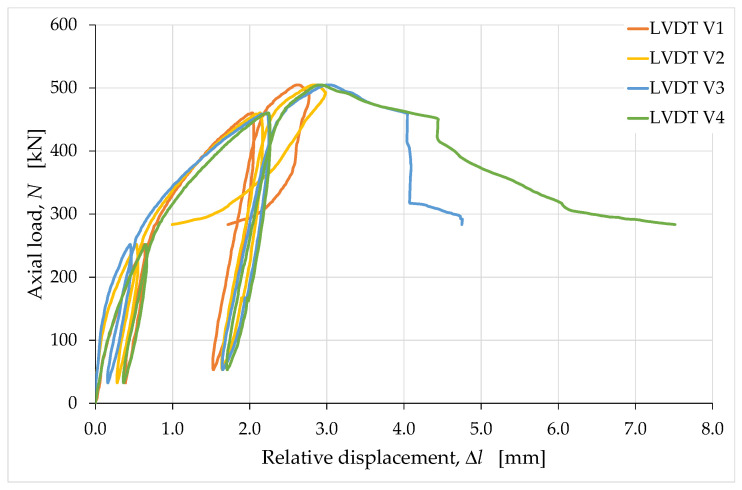
Load/displacement curves acquired along the vertical edges of the specimen.

The LVDTs positioned vertically, close to the vertical edges of the specimen, returned the load/displacement curves shown in Figure 9, where the displacements are actually relative displacements. Specifically, a positive displacement value in Figure 9 indicates that one end of the LVDT was approaching the opposite end, which caused the instrument to shorten.

The curves in Figure 9 diverge, starting from the maximum load value, which means that the upper face of the specimen underwent a significant rotation, starting from the peak load. In particular, the marked snap-back—Class II behavior of rock failure under uniaxial compression, with a reduction in displacement in the post-peak region [78]—of the curve acquired through LVDT V2 is indicative of an elongation of edge 2. This means that the upward displacement caused at the top of edge 2 by the rotation of the upper face (around the testing machine head) was prevalent over the downward displacement of the testing machine head. Edge 1 also underwent some elongation after the peak, albeit to a lesser extent, causing the curve acquired through LVDT V1 to snap back. In the opposite position to edges 1 and 2, the rotation of the upper face instead compressed edges 3 and 4, resulting in a further shortening of edges 3 and 4 with respect to that caused by the downward displacement of the head of the testing machine. This resulted in the Class I behavior of the curves acquired through LVDT V3 and LVDT V4, which means that these curves exhibited a monotonic increase in displacement after the peak. The reason for the rotation of the upper face was the failure of the short side face to the right of the operator.

**Figure 10:**
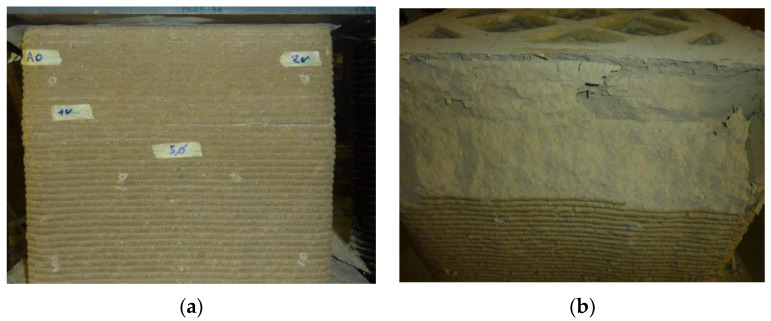
Failure mechanisms on the two short sides: (**a**) side located to the left of the operator, extending between edge 1 (left) and edge 2 (right); (**b**) side located to the right of the operator, extending between edge 3 (left) and edge 4 (right).

**Figure 11:**
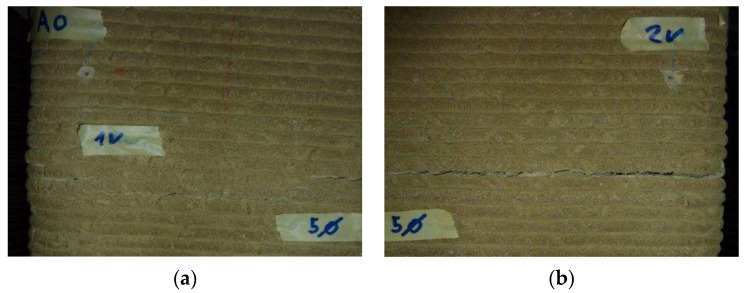
Details of the detachments between the horizontal layers of the side to the left of the operator: (**a**) left detachment, which reaches edge 1; (**b**) right detachment, which reaches edge 2.

Figure 10b shows the upper part of this side face, which underwent a detachment, with sliding along an inclined plane towards the outside. On the opposite side face, the extension due to the strong rotation of the upper face caused detachments between the horizontal layers (Figure 10a and Figure 11).

**Figure 12:**
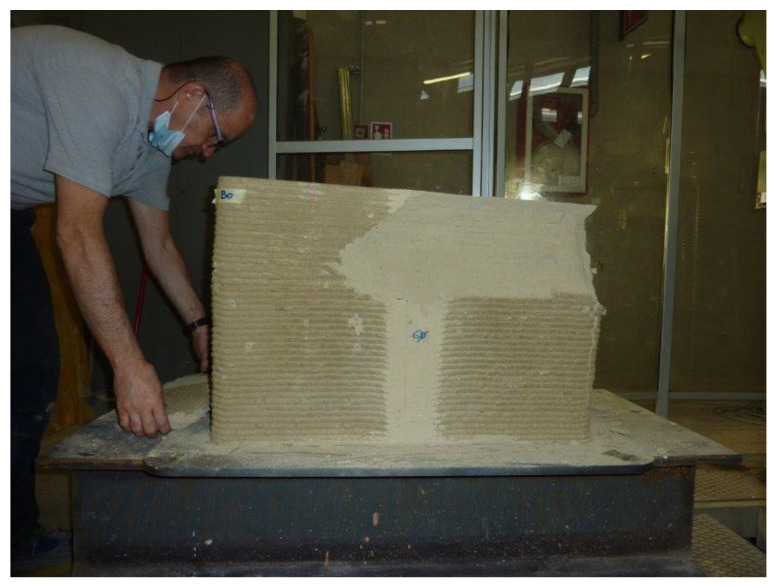
Front view of the specimen after the load test, with the upper face permanently inclined from left to right.

In particular, the opening of the right detachment (Figure 11b) was larger than the opening of the left detachment (Figure 11a) due to the greater elongation of edge 2 compared to edge 1. After the end of the load test and the removal of the specimen from the testing machine, the upper face retained a permanent value of rotation, with a left-to-right skew (Figure 12).

Due to the dependence on the rotation of the upper face, no curves among those acquired using the LVDTs V1, V2, V3 and V4 are representative of the axial displacement of the specimen. Consequently, none of them are useful for providing information on the rheology of the material undergoing uniaxial compression. Their average curve, on the other hand, provides the relationship between load and displacement for the points lying on the axis of the testing machine head. Since these points only moved down due to the displacement of the testing machine head, the average curve expresses a relationship that is unaffected by the effects of rotation. The average load/displacement curve is therefore suitable for identifying the stress/strain relationship representative of the actual uniaxial compression behavior.

**Figure 13:**
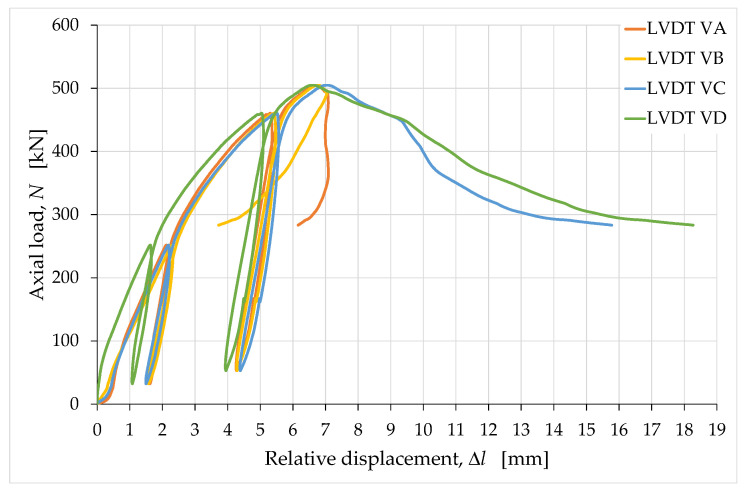
Load/displacement curves acquired between the corners of the metal plates for load distribution.

Similar considerations apply to the displacements acquired at the corners of the plates (the positive values of displacement in Figure 13 indicate both the absolute downward displacements of the corners of the upper plate and the relative displacements of mutual approach between opposite corners of the upper and lower plates).

In fact, the vertical LVDTs VA, VB, VC and VD also provided displacement values influenced by the rotation—of the upper plate, which lies on the upper face of the specimen—because they were located far from the axis of the specimen. In particular, the curve acquired via LVDT VB (near edge 2) displays a Class II behavior (Figure 13) as a consequence of the upward displacement of corner 2 (of the upper plate) caused by the rotation. At corner 1, the post-peak branch is, for the most part, almost vertical (curve acquired through LVDT VA in Figure 13). This means that the upward displacement caused by the rotation of the upper plate and the downward displacement caused by the movement of the testing machine head were almost perfectly balanced at corner 1. Therefore, corner 1 was close to the absolute rotation center of the upper plate. Finally, at corners 3 (near edge 3) and 4 (near edge 4), the rotation of the upper plate amplified the downwards displacements, which gave the curves acquired via LVDT VC and LVDT VD a Class I behavior (Figure 13).

In conclusion, even the curves showing the displacements acquired at the corners of the upper plate are not individually suitable for identifying the stress/strain relationship under uniaxial compression load, while their average curve is. Therefore, we used the average values of the (relative) displacements acquired along the edges of the specimen and at the corners of the plates—${\bar{\Delta l}}_{e}$ and ${\bar{\Delta l}}_{c}$, respectively—to identify the vertical strains along the axis of the specimen:

(5) $${\bar{\Delta l}}_{e}=\frac{1}{4}\left(\Delta l_{V1}+\Delta l_{V2}+\Delta l_{V3}+\Delta l_{V4}\right),$$

(6) $${\bar{\Delta l}}_{c}=\frac{1}{4}\left(\Delta l_{VA}+\Delta l_{VB}+\Delta l_{VC}+\Delta l_{VD}\right),$$

where $\Delta l_{V1}$, $\Delta l_{V2}$, $\Delta l_{V3}$, $\Delta l_{V4}$, $\Delta l_{VA}$, $\Delta l_{VB}$, $\Delta l_{VC}$ and $\Delta l_{VD}$ are the relative displacements (shortenings) acquired, respectively, by the LVDTs V1, V2, V3, V4, VA, VB, VC and VD.

The vertical strain $\varepsilon_{e}$ is then the ratio of ${\bar{\Delta l}}_{e}$ to the length of the LVDTs placed along the edges of the specimen, $l_{V_{e}}$, and $\varepsilon_{c}$ is the ratio of ${\bar{\Delta l}}_{c}$ to the length of the LVDTs placed between the corners of the upper and lower plates, $l_{V_{c}}$:

(7) $$\varepsilon_{e}=\frac{{\bar{\Delta l}}_{e}}{l_{V_{e}}},$$

(8) $$\varepsilon_{c}=\frac{{\bar{\Delta l}}_{c}}{l_{V_{c}}}.$$

**Figure 14:**
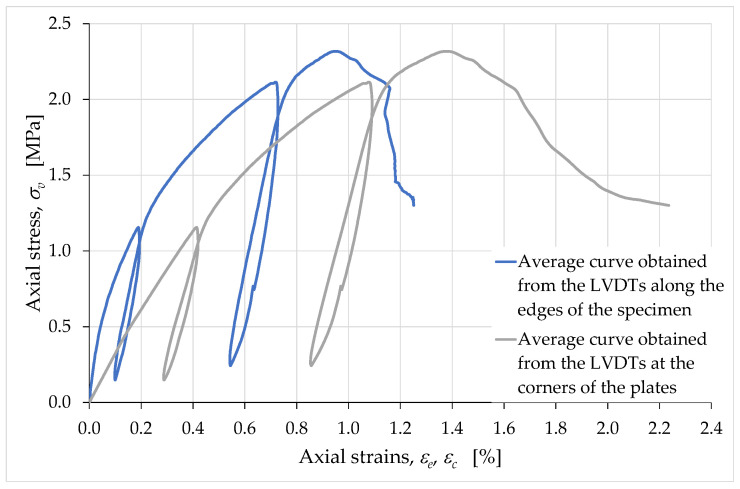
Average curves of the axial stress with respect to the axial strains $\varepsilon_{e}$ and $\varepsilon_{c}$.

Figure 14 shows the stress/strain curves obtained for $\varepsilon_{e}$ and $\varepsilon_{c}$ (positive strain values indicate shortening in the axial direction), with the axial stress, $\sigma_{v}$, given by the ratio between the axial load, N, and the extruded area (after shrinkage), $A_{extr}$:

(9) $$\sigma_{v}=\frac{N}{A_{extr}},$$

where $A_{extr}$ is the nominal area in Equation (4).

The greater deformability of the curve for $\varepsilon_{c}$ compared to the curve for $\varepsilon_{e}$ indicates that the deformation phenomena were more intense near the contact areas of the specimen with the external load. The localization of the strains close to the plates of the testing machine was particularly evident in the early stages of the load test, that is, near the origin of the diagrams in Figure 14. In fact, it is precisely near the origin that we can observe the greatest difference between the slopes of the two curves and, since these are stress/strain diagrams, their slopes represent stiffnesses. In the following sections, when we discuss the vertical strains, $\varepsilon_{v}$, we are referring to the values of the strains obtained from the average displacements at the corners.

(10) $$\varepsilon_{v}=\varepsilon_{c}.$$

If compared with the stress/strain diagrams of [58], Figure 14 provides information on the effects induced by the extrusion process on the mechanical properties of the mixture. In fact, the slopes in Figure 14 are monotonically nonincreasing functions up to the peak (excluding the unloading/reloading cycles), while the branches between the origins and the peaks in [58] have two points of inflection each, due to the closing of the voids during the compression test. Since the specimens in [58] were compacted by hand in the formworks, this means that the pressure to which the mixture is subjected in the extruder is able to close the voids in the mixture to a greater extent than manual compaction.

Figure 14 also provides indirect indications of the damage phenomena that develop inside the specimen during the compression test. In particular, this information derives from the average slopes of the unloading/reloading cycles [79], as their variation provides an empirical evaluation of the damage parameter (Figure S3). Since the average slope of unloading/reloading is almost the same for both cycles in Figure 14 (in both the $\varepsilon_{e}$ curve and the $\varepsilon_{c}$ curve), we can conclude that the damage effects were balanced by the opposite effects of the material compaction due to the increasing compressive load [58], at least as regards the growing branch of the stress/strain curves. It is worth noting that this balance of effects is peculiar to earthen mixtures and is not specific to the behavior of brittle construction materials. Being much less compressible than earthen mixtures, brittle construction materials have in fact unloading/reloading slopes that decrease as the load test proceeds (Figure S4). Therefore, the constancy of the unloading/reloading slopes is one of the results that characterize the earthen mixture as a material with hybrid characteristics, halfway between a soil and a properly labeled brittle material.

### 3.2. Behavior of the Horizontal Strains

**Figure 15:**
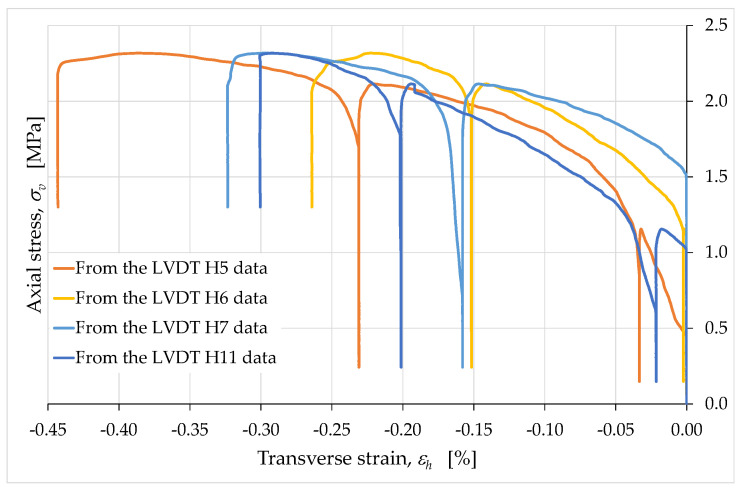
Axial stress/transverse strain curves acquired by the horizontal LVDTs between the ribs.

**Figure 16:**
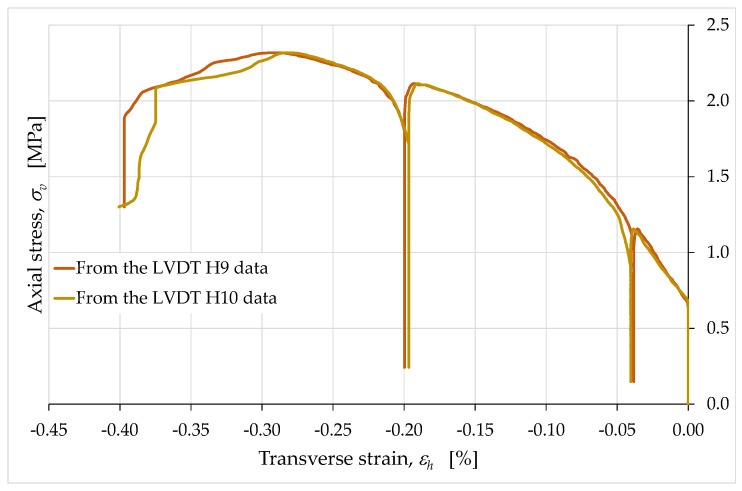
Axial stress/transverse strain curves acquired over the entire length of the long side faces.

**Figure 17:**
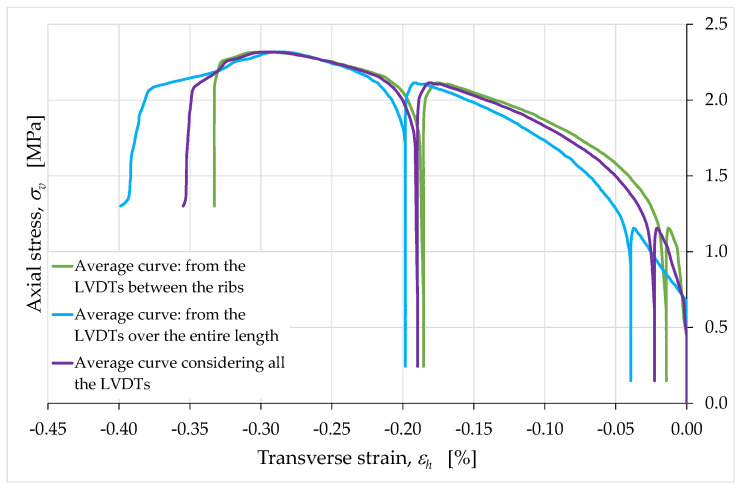
Average curves of axial stress with respect to transverse strain, obtained from the relative displacements over the inter-rib length and over the entire length of the long side faces.

**Figure 18:**
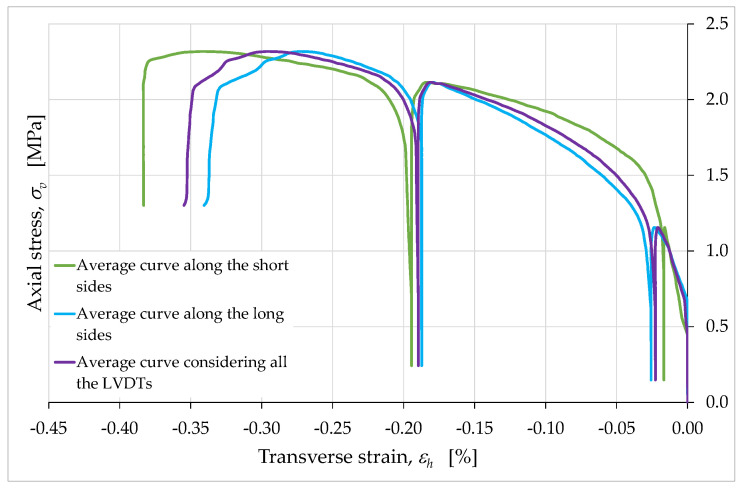
Average curves of axial stress with respect to transverse strain, obtained from the relative displacements over the short and the long side faces.

**Figure 19:**
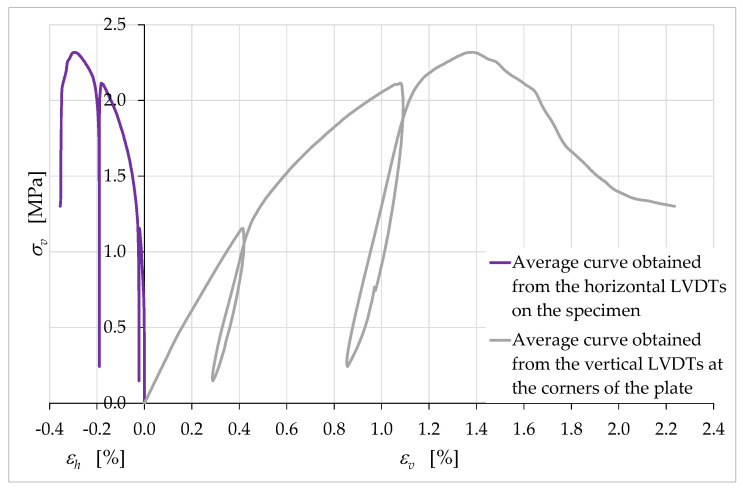
Average curves of axial stress with respect to axial and transverse strain.

The relationship between the horizontal and vertical strains turned out to be that which is typical of solid mechanics, characterized by elongations (negative strains in Figure 15, Figure 16, Figure 17, Figure 18 and Figure 19) along the directions orthogonal to that of the monoaxial compressive load (the Poisson effect).

The LVDTs placed horizontally allowed us to draw more than one conclusion about the horizontal strains, which turned out to be very small in all cases (in Figure 15, Figure 16, Figure 17, Figure 18 and Figure 19, we excluded the data acquired by LVDT H8, due to instrumental errors). The first observation we want to focus on concerns the comparison between the horizontal strains estimated over the inter-rib length of the ribs described in Section 2.3 (Figure 15) and the horizontal strains estimated over the entire length of the side faces (Figure 16). This comparison does not reveal evident localization phenomena of the horizontal strains, since the average curve obtained over the inter-rib length differs by minimal quantities from the average curve obtained over the entire length of the side faces (Figure 17).

Therefore, the presence of an infill did not have a significant effect on the strains of the external envelope. In other words, despite the presence of hollow areas inside the specimen, the overall behavior was that typical of a solid without cavities. In particular, the almost complete superimposition of the two curves of Figure 16 shows that the two long side faces (one in front of the operator and one on the opposite side) underwent the same deformation history.

The average curves obtained for the long sides (from the values of the LVDTs H6, H9, H10 and H11) and for the short sides (from the values of the LVDTs H5 and H7) confirm the conclusions about the role of the internal infill (Figure 18). In fact, even in the latter case the difference between the two average curves is minimal.

We can therefore assume that the average strain along the horizontal direction of the long side faces, $\varepsilon_{h_{1}}$, was equal to the average strain along the horizontal direction of the short side faces, $\varepsilon_{h_{2}}$:

(11) $$\varepsilon_{h_{1}}=\frac{1}{4}\left(\frac{\Delta l_{H6}}{l_{H6}}+\frac{\Delta l_{H9}}{l_{H9}}+\frac{\Delta l_{H10}}{l_{H10}}+\frac{\Delta l_{H11}}{l_{H11}}\right)=\frac{1}{4}\left(\frac{\Delta l_{H6}+\Delta l_{H11}}{l_{H_{r}}}+\frac{\Delta l_{H9}+\Delta l_{H10}}{l_{H_{s}}}\right),$$

(12) $$\varepsilon_{h_{2}}=\frac{1}{2}\left(\frac{\Delta l_{H5}}{l_{H5}}+\frac{\Delta l_{H7}}{l_{H7}}\right)=\frac{1}{2}\frac{\Delta l_{H5}+\Delta l_{H7}}{l_{H_{r}}},$$

(13) $$\varepsilon_{h_{1}}=\varepsilon_{h_{2}}=\varepsilon_{h},$$

where:

- $\Delta l_{H5}$ is the elongation of LVDT H5;
- $\Delta l_{H6}$ is the elongation of LVDT H6;
- $\Delta l_{H7}$ is the elongation of LVDT H7;
- $\Delta l_{H9}$ is the elongation of LVDT H9;
- $\Delta l_{H10}$ is the elongation of LVDT H10;
- $\Delta l_{H11}$ is the elongation of LVDT H11;
- $l_{H5}=l_{H6}=l_{H7}=l_{H11}=l_{H_{r}}$ is the length of the LVDTs between the ribs;
- $l_{H9}=l_{H10}=l_{H_{s}}$ is the length of the LVDTs that extend along the entire long side faces.

The equalities in Equation (13) mean that the specimen was transversely isotropic, that is, isotropic in the cross-section.

The peculiarity of the horizontal strain curves in Figure 15, Figure 16, Figure 17 and Figure 18 concerns the behavior during the unloading/reloading cycles. In fact, unlike what happened for the vertical strains, the unloading/reloading cycles in Figure 15, Figure 16, Figure 17 and Figure 18 do not show a hysteretic behavior and follow a vertical path. This highlights a clear difference in behavior between the horizontal strains, $\varepsilon_{h}$, and the vertical strains, $\varepsilon_{v}$ (Figure 19).

In particular, the absence of a hysteretic behavior for $\varepsilon_{h}$ indicates the nonoccurrence of dissipative effects along the horizontal direction (while they occurred along the vertical direction). The vertical path of the $\varepsilon_{h}$ cycles, on the other hand, provides information on the elastic and plastic components of the horizontal strain. In fact, since the elastic strain is the strain recovered at the unloading, we can conclude that the strain that occurred in the horizontal direction had only a plastic component (not recoverable at the unloading). Since the horizontal direction was also the extrusion direction, it therefore appears that the extrusion process had a decisive effect on the rheology of the extruded material, imparting only the plastic component to the strains along the extrusion direction. Along the direction orthogonal to that of the extruded layers, however, the strains showed both the plastic and the elastic component (Figure 19).

We can therefore speak of anisotropy induced by the extrusion process. To be precise, since the specimen was transversely isotropic due to Equation (13), the overall behavior of the specimen was orthotropic. The main consequence of the orthotropy induced by the extrusion process was the invariance of the linear dimensions of the cross-section at the removal of the axial load.

### 3.3. Relationships between Strains

Figure 19 also provides information on the ratio between the horizontal strains, $\varepsilon_{h}$, and the vertical strains, $\varepsilon_{v}$. In continuum mechanics, the negative of this ratio gives the value of Poisson’s ratio, ν, which is the negative of the ratio of transverse strain to axial strain:

(14) $$\nu =-\frac{\varepsilon_{h}}{\varepsilon_{v}}.$$

Strictly speaking, in the mechanical testing it is incorrect to continue to give the ratio in Equation (14) the name of Poisson’s ratio, for at least two reasons. The first reason is that Poisson’s ratio is one of the elastic coefficients that define Hooke’s law in a linear elastic regime, while the loads of the uniaxial compression test led the material well beyond its own range of linear elastic behavior. The second reason is that the macrocracks that propagated inside the specimen during the test (Figure S5a,b) do not allow the treatment of the specimen as a continuum [80], making it impossible to use the definitions of continuum mechanics. For the same reason, it is also necessary to revise the definitions of $\varepsilon_{h}$ and $\varepsilon_{v}$, which lose the meaning they have in continuum mechanics (deformations at a given point). In analogy to the definition of engineering strains, they are the ratios of the relative displacements (between points) to the reference lengths. During mechanical tests, however, the (relative) displacements between points can occur due to both the deformation of the body (rheological behavior) and the opening of cracks (nonrheological behavior), which results from the rigid body motions of the new surfaces generated by the cracks [81]. This deprives the ratio between the engineering strains of rheological significance. Therefore, we will continue to refer to the ratio ν in Equation (14) as the negative of the ratio of the (engineering) strains along two orthogonal directions, avoiding calling it Poisson’s ratio.

Due to the small values of $\varepsilon_{h}$ until the end of the load test, ν maintained unusually low values for a brittle construction material. As an example, Table 4 provides the values of ν for a few instants of the uniaxial compression test on the wall segment.

The trend of the values in Table 4 is very unusual for a brittle construction material. In fact, ν first increases and subsequently decreases, while in brittle construction materials it increases for the entire duration of the uniaxial compression test (Figure S6: curve with the transverse strain acquired on the external surface, by means of the strain gauge of Figure S7a). Furthermore, it is worth remembering that values of ν close to zero are characteristic of open-cell polymer foams, due to the tendency of the cells to collapse under compression [82]. Therefore, such low values of ν in Table 4 suggest that the extruded earthen mixture had a high number of voids that tended to collapse during the compression test. According to the curve of the horizontal strains in Figure 19 and the values of ν in Table 4, in the case of the earthen mixture, the voids tended to collapse mainly at the beginning of the compression test and after the peak of the stress/strain curve. As for the reason for the presence of such a large number of voids, it may have been caused by both the high percentage of soil in the mixture and the layer-by-layer extrusion process.

**Figure 20:**
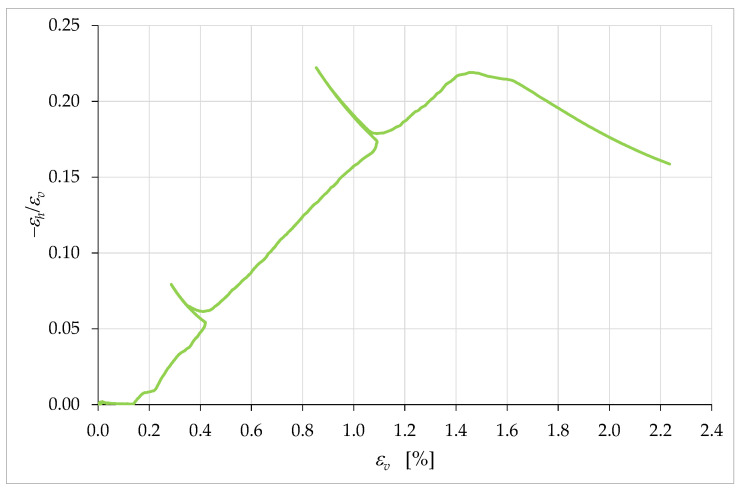
Curve of ν (apparent Poisson’s ratio) with respect to the axial strain, $\varepsilon_{v}$.

Figure 20, which is the graph of the values of ν versus the axial strain, $\varepsilon_{v}$, shows that the increase in ν ended at $\varepsilon_{v}={\hat{\varepsilon }}_{v}$, which was the strain at the maximum stress of the stress/strain curve, $\sigma_{v_{max}}$ (Figure 19):

(15) $${\hat{\varepsilon }}_{v}={\varepsilon_{v}|}_{\sigma_{v}=\sigma_{v_{max}}}≅1.4\%.$$

Then, ν assumed an almost constant behavior at first and, subsequently, decidedly decreased. It is worth noting that the last point of the curve in Figure 20 is not indicative of the actual failure of the specimen. In fact, since the LVDTs showed a tendency to detach from the specimen in the final stages of the test, it was preferred to interrupt the acquisition of the displacements and remove the LVDTs in advance, to protect the instruments from damage due to a fall. Therefore, the curve actually continues beyond the last acquisition point. The slope of the curve at the last point suggests that the final value of ν could be significantly lower than the last identified value, equal to 0.159 (Table 4).

Figure 20 also shows near-zero values of ν for the linear elastic regime, which indicates the tendency of the specimen to collapse on itself without lateral expansion. Both the decreasing behavior after the maximum load and the values close to zero in the linear elastic regime are absolute novelties for the ratio ν of a brittle construction material. As with the collapse of voids, these behaviors could have derived from the properties of the soil that made up a large part of the mixture.

**Figure 21:**
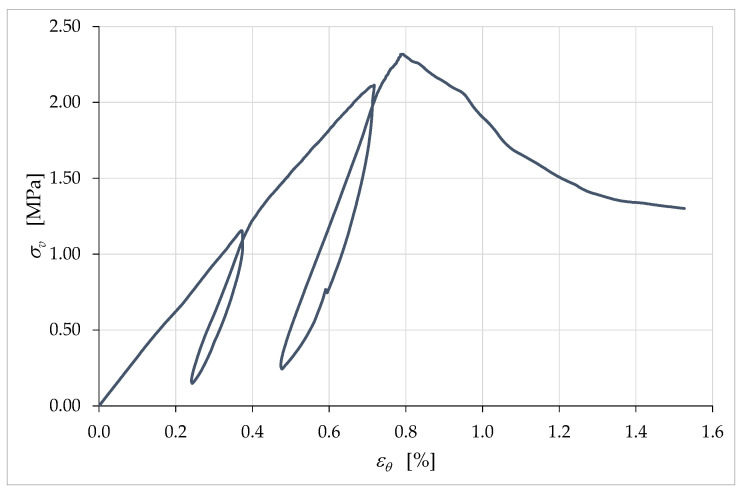
Curve of the axial stress, $\sigma_{v}$, with respect to $\varepsilon_{\vartheta }$ (apparent volumetric strain).

Equally surprising for a brittle construction material is the sum of the strains along the three orthogonal directions of the axis and the two horizontal sides (Figure 21):

(16) $$\varepsilon_{\theta }=\varepsilon_{v}+\varepsilon_{h_{1}}+\varepsilon_{h_{2}}=\varepsilon_{v}+2\varepsilon_{h},$$

where we made use of Equation (13).

In continuum mechanics, $\varepsilon_{\theta }$ is the volumetric strain, that is, the ratio of the change in volume of a body, ΔV, to the original volume of the body, V:

(17) $$\varepsilon_{\theta }=\frac{\Delta V}{V}.$$

Due to crack propagation, however, the rigid body motions of the crack surfaces also affect the sums in Equation (16) [83]. Consequently, like ν, $\varepsilon_{\theta }$ also has no real rheological significance in a mechanical test, but is indicative of an average behavior at the mesoscale.

Between the origin and the peak (where $\sigma_{v}=\sigma_{v_{max}}$), the values of $\varepsilon_{\theta }$ in Figure 21 are very close to being directly proportional to the axial stress, $\sigma_{v}$ (the positive values of $\varepsilon_{\theta }$ in Figure 21 imply a decrease in volume). This does not correspond to the behavior of $\varepsilon_{\theta }$ for brittle construction materials, such as concrete (Figure S8: curve with the transverse strain acquired on the external surface, by means of the strain gauge in Figure S7a). Indeed, the $\varepsilon_{\theta }$ curve between the origin and the peak of brittle construction materials changes from being concave (concave downward) to convex (concave upward).

The nearly linear behavior up to the peak in Figure 21 makes K, the ratio between $\sigma_{v}$ and $\varepsilon_{\theta }$, almost constant up to the peak:

(18) $$K=\frac{\sigma_{v}}{\varepsilon_{\theta }}.$$

**Figure 22:**
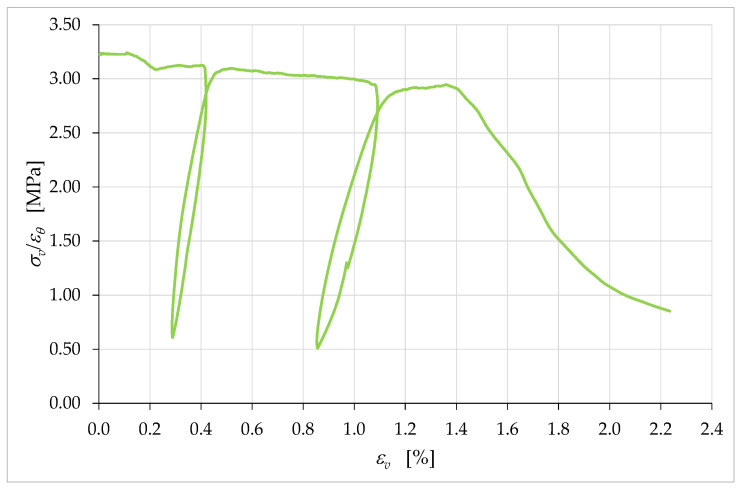
Dependence of the ratio K on the axial strain, $\varepsilon_{v}$.

In Figure 22, this means that K is almost constant in the range $0\leq \varepsilon_{v}\leq {\hat{\varepsilon }}_{v}$, with ${\hat{\varepsilon }}_{v}$ defined in Equation (15).

Continuing the comparison between the behavior of brittle materials and earthen mixtures, the convex branch of the $\varepsilon_{\theta }$ curve of brittle materials assumes a vertical tangent for a stress value less than the maximum stress (Figure S8: curve with the transverse strain acquired on the external surface). Subsequently, the values of $\varepsilon_{\theta }$ begin to decrease so much that they change the sign, and the curve becomes concave again (Figure S8: curve with the transverse strain acquired on the external surface). The change in the sign of $\varepsilon_{\theta }$ indicates an increase in the volume of brittle specimens, which reach a final volume greater than the original volume (dilative behavior) [83,84,85]. In Figure 21, on the contrary, the curve $\varepsilon_{\theta }$ never assumes a vertical tangent and $\varepsilon_{\theta }$ is an ever-increasing function. This means that the earthen specimen displayed contractive behavior until the end of the test, while the brittle materials become dilative after the maximum load.

Obviously, all of the above is valid within the limits of the two components, one rheological and one not, which make up the (relative) displacements acquired on the external surface of the specimen. It is worth noting that, if we identify the strains by eliminating the non-rheological components [83], $\varepsilon_{\theta }$ is an ever-increasing function (in absolute value) even in brittle construction materials (Figure S8: curve with the transverse strain acquired inside the resistant structure, by means of the fiberoptic sensor in Figure S7b). Even the values of ν change in the latter case, causing the $\nu /\varepsilon_{v}$ curve of brittle construction materials to decrease (in absolute value) in the final branch (Figure S6: curve with the transverse strain acquired inside the resistant structure, by means of the fiberoptic sensor in Figure S7b). We can therefore interpret the ever-increasing behavior of $\varepsilon_{\theta }$ in Figure 21 and the negative slope of the last branch of the $\nu /\varepsilon_{v}$ curve in Figure 20 as indications that, in earthen materials, the nonrheological component of the displacement is negligible compared to the rheological component. This means that earthen materials under uniaxial compression loads tend to develop far fewer cracks than brittle construction materials, which confirms the conclusions we had reached based on the slope of the unloading/reloading cycles in Figure 14. In conclusion, the analysis of the strains developed during a uniaxial compression test confirms that the extrusion process of earthen mixtures results in a material with peculiar mechanical properties—at the mesoscale—halfway between those of a brittle material and a soil.

### 3.4. Failure Mechanisms and Compressive Strength

**Figure 23:**
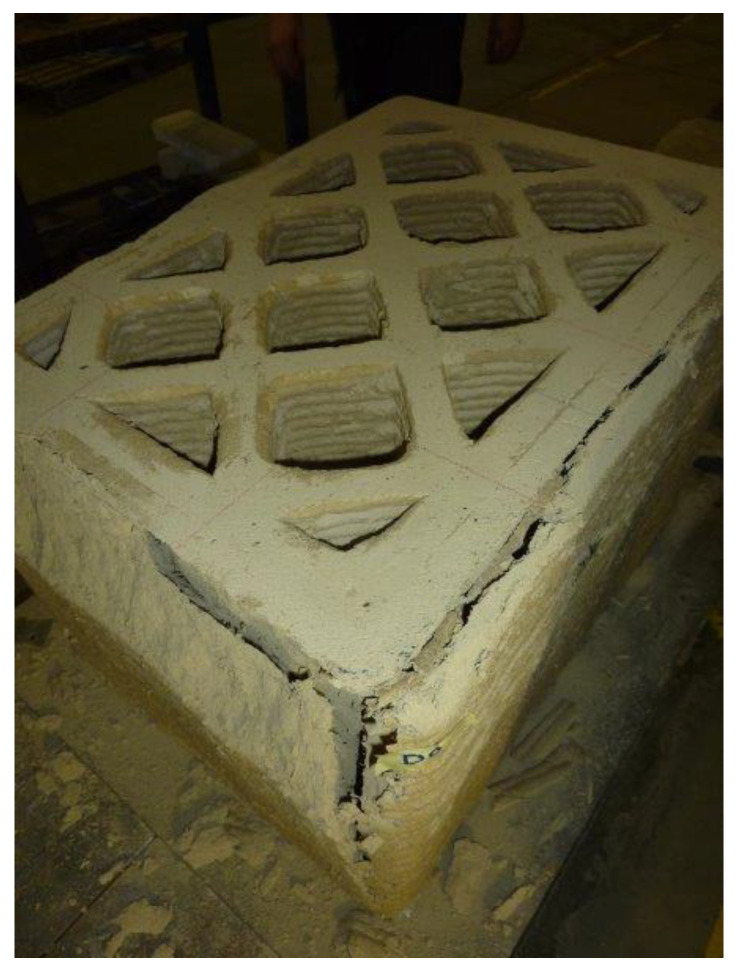
Appearance of the specimen after raising the head of the testing machine.

The failure of the specimen began at the contact edges between the specimen and the plates, with the enucleation of inclined sliding planes that propagated towards the interior of the specimen. This led to the specimen cracking along the vertical edges and the outward ejection of the outermost part of the specimen (Figure 23). However, there was no real collapse of the external part, not even at the end of the load test (Figure 23).

**Figure 24:**
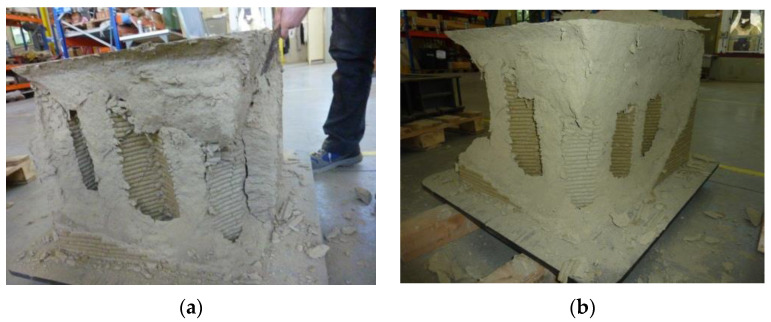
Side views of the specimen after the test: (**a**) before and (**b**) after the removal of the material isolated (only in part) by the sliding surfaces.

**Figure 25:**
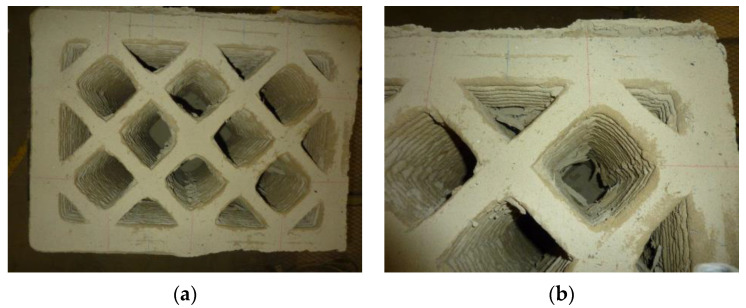
Top view of the specimen after the test: (**a**) overall view and (**b**) detail of the corner at the top right.

The manual removal of the external cladding material (Figure 24a)—isolated only in part by the sliding surfaces—highlighted the double truncated pyramidal structure of the internal part (Figure 24b), which retained a fair capacity to withstand loads. This failure mechanism is similar to that observed in cubic and cylindrical concrete specimens when subjected to uniaxial compression tests (Figure S5). It is worth noting, however, that, unlike what happens in concrete, the damaging mechanism of the earthen specimen affected only the outermost part, stopping at the internal infill. In fact, it seems that the honeycomb structure of the infill was able to interrupt the propagation of the macrocracks towards the inside, preserving the infill itself from the main damage mechanisms (Figure 25a). Only minor crushing damage, mainly located near the external contour, affected the internal infill (Figure 25b).

**Figure 26:**
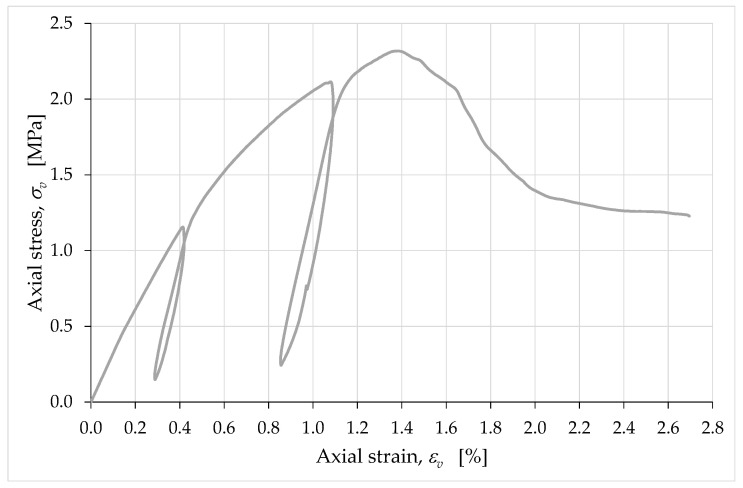
Complete average curve of the axial stress with respect to the axial strain, obtained from the acquisitions of the LVDTs placed between the corners of the metal plates for the distribution of the load.

The crack-stopping action exerted by the infill was also decisive in determining the shape of the stress/strain curve. The $\sigma_{v}/\varepsilon_{v}$ curve in Figure 26 differs from the $\sigma_{v}/\varepsilon_{c}$ curve in Figure 14 only in the range of displayed data. In fact, as previously mentioned, it was preferred to remove the LVDTs installed on the surface of the specimen in advance, to avoid damaging the instruments. For the purpose of comparison, in Figure 14 this resulted in truncating the curve $\sigma_{v}/\varepsilon_{c}$ at the instant of the last LVDT acquisition on the specimen surface. Since the LVDTs placed between the corners of the plates did not suffer any danger of damage, they actually continued to acquire the displacements until the end of the compression test. Figure 26, therefore, shows the stress/strain curve obtained from the acquisitions at the corners up to the test end, which did not coincide with the failure of the specimen. The interruption of the test was in fact necessary anyway, to avoid the LVDTs at the corners of the plates going out of scale.

The larger set of data in Figure 26 allows us to appreciate the shape of the final part of the $\sigma_{v}/\varepsilon_{v}$ curve, which tends asymptotically to a horizontal plateau. When compared with the stress/strain curves of brittle construction materials (Figure S9), even this plateau exhibits unusual behavior, although brittle construction materials may also have asymptotic final behavior. In fact, the softening branch in Figure 26 seems to find an asymptotic limit for a fraction of the maximum stress significantly greater than that typical of brittle construction materials (strain-softening is the decline of uniaxial stress with increasing strain [86]). To give an idea of the order of magnitude of the quantities involved, the ultimate stress in Figure 26 is approximately 53% of the maximum stress, while the ultimate stress of brittle construction materials is close to zero.

The cause of this anomalous behavior is not the biostabilizing effect of the RH, as previous studies on cast specimens made with biostabilized earthen mixtures have never shown a high residual stress plateau [61,87]. Rather, the high value of ultimate stress (as a percentage of the maximum stress) can find an explanation in the crack-stopping action of the infill. Indeed, experimental [80] and numerical [88] studies on the shape effect of brittle solids have shown that the softening branch is steeper when the damage phenomena are more developed. Consequently, the softening branch provides indirect information on the resistant cross-section, because the area of the resistant cross-section (resistant area)—which decreases faster when the damage phenomena develop faster (Figure S3)—decreases faster even when the softening branch is steeper [89]. Therefore, asymptotic behavior with a high value of residual stress is symptomatic of a crack pattern that only partially affects the resistant cross-section. In the specific case of a 3D-printed wall segment, it is also a proof of the effectiveness of the infill in blocking the propagation of cracks. This gives a physical meaning to the final plateau of the softening branch, allowing us to state that the higher the plateau the greater the resistant area at the end of the test. Thus, the shape of the curve in Figure 26 reveals that a 3D-printed specimen made of earthen material retains a greater resistant area than an identical specimen made of brittle construction material.

As far as strength is concerned, the maximum stress in Figure 26 is equal to 2.32 MPa. This value falls within the dispersion range of the strength at 90 days of curing evaluated on cubic specimens made with the same earthen mixture and compacted by hand in the formworks [58], with an average value of 2.50 MPa. This means that, along the direction orthogonal to the layers, the layer-by-layer extrusion process did not substantially change the strength of the earthen material compared to that of the hand-compacted material. In other words, the 3D-printing process was good enough to avoid decay in the mechanical strength properties due to imperfect cohesion between the superimposed layers.

**Figure 27:**
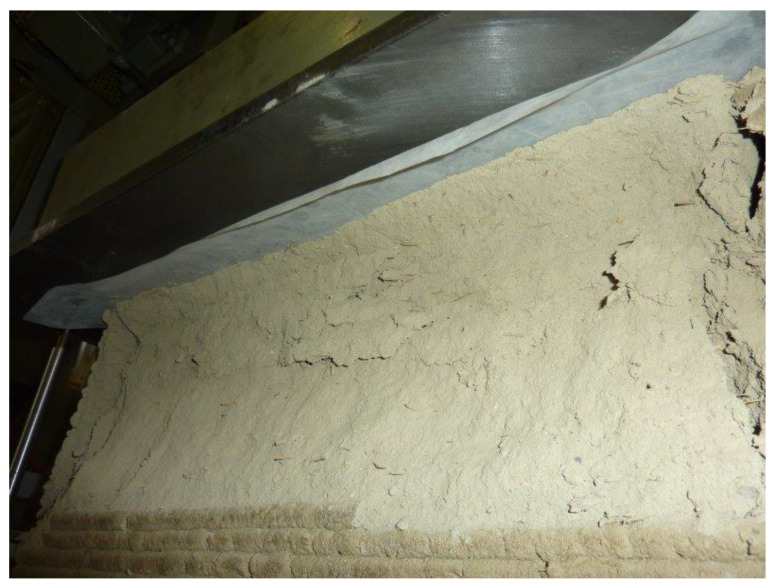
Appearance of the extruded layers along one of the sliding surfaces activated by crack propagation.

The good cohesion between the extruded layers is also evident in Figure 27, which shows a sliding plane that intersects several layers in the outer envelope. In Figure 27, in fact, there are no discontinuities evident between the layers, nor are there any detachments between the two shells that make up the external envelope (Figure 1). On the other hand, the external part of the specimen can develop the same sliding planes of a homogenous material, cast in the formwork, precisely because of the good cohesion between its extruded layers.

The compressive strength value of 2.32 MPa is a good result for a 3D-printed load-bearing earthen element, as it is slightly higher than the minimum compressive strength required for one-story external walls of rammed earth (2 MPa) [36].

## 4. Discussion

The main motivation of the experimental test presented in this paper was to investigate the major criticalities under a uniaxial compression load of a 3D-printed wall segment made with an earthen mixture, in view of possible uses for the construction of multi-story buildings. The results of the compression test showed that the two possible criticalities identified a priori—namely, the low degree of collaboration between the layers and the overturning of the external envelope—do not actually constitute real dangers for the stability of the earthen structure under vertical loads. As far as the first criticality is concerned, in fact, the layers showed sufficient cohesion to resist the loads as a single rigid body, so much so that the sliding surfaces showed the same peculiarities of compact materials and the strength was comparable to that of cubic specimens made with the same mixture. Regarding the second criticality, the load test actually showed a tendency towards the detachment of the lateral faces, but the very small Poisson’s ratio kept the outward displacements so low that they did not to cause overturning problems. Rather, the specimen showed a tendency to implosion, especially at the beginning and end of the load test.

### 4.1. Main Findings

The experimental results made it possible to obtain more information than we had set out to acquire from this experimental test, that is, the verification of the cohesion between layers and the sensitivity to overturning of the lateral faces. Some of this information is particularly important because it characterizes the earthen mixture as a material that does not fit perfectly into the class of brittle construction materials; therefore, it is deserving of a separate classification. In fact, if studied at the mesoscale, the earthen mixture provides values of (apparent) Poisson’s ratio (ν) and (apparent) volumetric strain ($\varepsilon_{\theta }$) that have little to do with the homologous quantities of brittle construction materials. The major anomalies with respect to the typical mechanical behavior of brittle construction materials consist of:

- Values close to zero of ν in the linear elastic regime (implosive behavior);
- Low values of ν for the entire duration of the compression test;
- Decreasing values of ν in the final part of the compression test;
- (Almost) Direct proportionality between $\varepsilon_{\theta }$ and the axial stress, $\sigma_{v}$, up to the maximum load;
- Increasing values of $\varepsilon_{\theta }$ (contractive behavior) until the end of the compression test;
- Stress/strain curve with asymptotic behavior towards an ultimate stress value equal to about 50% of the maximum stress.

From the comparison with the studies for the experimental identification of the damage parameter in concrete solids, it emerged that all these anomalies, apart from the first one, depended on the ability of the internal infill to stop the propagation of macrocracks inside the specimen. This has direct repercussions on the value of the resistant area, which decreases much more slowly in the earthen solid than in a full solid of identical external dimensions made of concrete. From the point of view of identifying the constitutive relationship, this means that the difference between mesoscale and microscale behavior is much less marked for earthen material than it is for concrete.

By reversing the point of view, the crack-stopping action of the internal infill can find applications for design purposes. In other words, it is possible to give the resistant area of the 3D-printed load-bearing earthen elements characteristics as similar as possible to any specific desired behavior, simply by adequately designing the internal infill. This gives the internal infill a real structural function.

As far as the first anomaly is concerned, it is likely that it depends on the presence of a high percentage of voids, which tend to collapse during the compression test. Even the results of a previous experimental campaign on the strength of cubic specimens compacted in formwork showed that the earthen mixture has a high percentage of voids. The value of ν close to zero in the linear elastic regime is therefore a rheological property, which depends on the granulometric melt of the mixture. Specifically, it depends on the fractions of the mixture that have the greater grain size and on the low percentage of fine fractions.

From this experimental test, a further result emerged, namely, the orthotropic behavior of the earthen specimen. In particular, the behavior turned out to be isotropic in the cross-section but different from the behavior along the axial direction. The reason for the orthotropy lies in the changes to the rheology of the mixture that followed from the extrusion process. The high pressure undergone by the mixture during extrusion, in fact, seems to have plasticized the mixture along the direction of extrusion. The plasticization was such that the displacements in the cross-section were not at all recoverable upon the unloading. Along the axial direction, however, the displacements also had an elastic component, which were recoverable at unloading.

### 4.2. Limitations of the Study

Usually, uniaxial compression tests conducted on specimens in the laboratory only provide a partial view of the behavior of the material in situ, as they do not take into account the confinement effect exerted between contiguous elements. In other words, a wall can be seen as a succession of vertical segments, each of which limits the free lateral expansion of the continuous segments by imparting horizontal forces that modify the overall compressive strength (due to the Poisson effect). Since the uniaxial compression test does not exert horizontal forces on the specimen, this test is not therefore representative of the actual behavior of the material in situ. A further reason why the uniaxial compression test is not representative of the actual behavior is the development of inclined sliding planes, which cause a decrease in the resistant area of the specimen.

In the specific case of the 3D-printed earthen wall segment, however, the behavior under uniaxial load of the specimen was reasonably representative of the load-bearing capacities of the entire wall. In fact, the value of Poisson’s ratio close to zero makes the interactions between the contiguous vertical elements very low and the crack-stopping action of the infill allows the resistant area to differ slightly from the nominal area.

Rather, the reasons for uncertainty concerning the experimental results lie in the environmental conditions. Both the 3D-printing process and the curing of the wall segment took place in the laboratory, under controlled thermohygrometric conditions. This was important for the replicability of the experimental results, but it is also the major limitation of this study. Indeed, in the spirit of a zero-kilometer construction, WASP’s 3D-printing technology consists of the onsite printing of buildings, with the transportation of the 3D printer to the construction site. Since the thermohygrometric conditions of the construction site depend on the local environmental conditions, the printing process of a building can also take place in conditions that are very different from those controlled in the laboratory. This has an impact primarily on the volume of mixing water. For example, printing in very hot climates may require more mixing water to counteract the decay of fluidity due to strong evaporation. Since the compressive strength depends on the volume of mixing water, the local environmental conditions at the time of printing have therefore an impact on the compressive strength of the building. In addition, as the local temperature and humidity vary continuously over the time span of the printing process—which takes a few days of work—the volume of mixing water requires continuous recalibration over the course of a printing day. This may result in different compressive strengths for the building portions printed under different thermohygrometric conditions. Therefore, in addition to the orthotropy caused by the extrusion process (Section 3.2), earthen buildings printed onsite may suffer from an anisotropic effect to a degree that is not known a priori, due to the changing environmental conditions of the construction site.

To verify the actual impact of the environmental conditions on the compressive strengths, it may be appropriate to subject to uniaxial compression testing and compare the results of several mixture samples taken from the muller at different times during the onsite printing process. As a general recommendation, it is in any case advisable to try to protect the printing site as much as possible from thermal excursions, in order to limit the variability of the compressive strengths in the building.

## 5. Conclusions

This paper is part of the research on the 3D printing of earthen housing modules made with soil excavated in situ. Previous studies have already led to the definition of 3D-printed earthen elements for the external cladding of single-story wooden load-bearing structures. With this work, we intended to take a step forward in the use of 3D-printed earthen elements, studying their load-bearing capacity under vertical loads. The compressive strength of the 3D-printed earthen elements turned out to be comparable to the compressive strength of traditional rammed-earth buildings.

Since the ultimate goal of this line of research is to build load-bearing structures entirely from earth, consisting of two or more floors, the present work investigated two of the major alleged criticalities of 3D-printed multi-story earthen buildings: detachments due to poor cohesion between the extruded layers and detachments between the internal infill and external shell. The uniaxial compression test did not actually show any particular danger for the stability of the structure due to detachment phenomena. Rather, the experimental results showed some mechanical behaviors that were completely unexpected, as they are quite anomalous for a brittle construction material. Among these, the most important were the near-zero value of the (apparent) Poisson’s ratio in the elastic regime and the contractive behavior until the end of the test. The causes of these two anomalous behaviors had different origins, rheological for the first and structural for the second. In fact, the contractive behavior (at the mesoscale) was the result of a poor propagation of cracks, due to the crack-stopping action of the infill. This means that the infill of the 3D-printed elements has not only a filling function, but also a real structural function.

The crack-stopping action of the infill was, then, the cause of several other anomalous behaviors of the 3D-printed element made with earthen mixtures, such as the high values of the resistant area of the cross-section and the low values of the Poisson’s ratio until the end of the uniaxial compression test.

## Figures and Tables

**Table 1:** Percentage composition by weight of the LT mix.

Component	Percentage by Weight
Soil	70.42%
Lime-based binder	4.70%
Hydraulic lime	4.69%
Shredded rice husk	1.41%
Silica sand	18.78%

**Table 2:** Main linear dimensions of the specimen in the hardened state, after shrinkage.

Linear Dimension	Value Reached in the Hardened State
Length	710.0 mm
Depth measured along the axis of symmetry	533.0 mm
Minimum depth between opposite ripples	515.0 mm
Height	490.0 mm
Thickness of the linear sections of the infill	35.5 mm
Thickness of the external cladding	66.0 mm

**Table 3:** Labelling and positioning of the 15 LVDTs: opposite corners of the upper and lower plates have the same numbering.

Label	Direction of Acquisition	Positioning
LVDT V1	Vertical	Near vertical edge 1, on the short side face to the left of the operator
LVDT V2	Vertical	Near vertical edge 2, on the short side face to the left of the operator
LVDT V3	Vertical	Near vertical edge 3, on the short side face to the right of the operator
LVDT V4	Vertical	Near vertical edge 4, on the short side face to the right of the operator
LVDT VA	Vertical	Between corner 1 of the upper plate and corner 1 of the lower plate
LVDT VB	Vertical	Between corner 2 of the upper plate and corner 2 of the lower plate
LVDT VC	Vertical	Between corner 3 of the upper plate and corner 3 of the lower plate
LVDT VD	Vertical	Between corner 4 of the upper plate and corner 4 of the lower plate
LVDT H5	Horizontal	Between the 2 ribs of the short side face to the left of the operator
LVDT H6	Horizontal	Between 2 of the ribs of the long side face in front of the operator
LVDT H7	Horizontal	Between the 2 ribs of the short side face to the right of the operator
LVDT H8	Horizontal	Between 2 of the ribs of the long side face on the opposite side of the operator
LVDT H9	Horizontal	Along the entire length of the long side face in front of the operator
LVDT H10	Horizontal	Along the entire length of the long side face on the opposite side of the operator
LVDT H11	Horizontal	Between 2 of the ribs of the long side face on the opposite side of the operator

**Table 4:** Some of the values assumed by the coefficient ν during the test.

Stress Value	ν
Half the stress of first unloading	0.008
Stress at the beginning of the first unloading/reloading cycle	0.051
Stress at the beginning of the second unloading/reloading cycle	0.168
Maximum stress	0.214
Ultimate stress	0.159

## Data Availability

Not applicable.

## Supplementary Materials

The following are available online at https://www.mdpi.com/article/10.3390/ma15020438/s1, Figure S1: the Gaia House by WASP—first prototype of a 3D-printed earthen building with biodegradable materials (built in 2018) (source: WASP), Figure S2: the Tecla House by WASP—3D printing of the second prototype of an earthen building (completed in 2021) (source: WASP), Figure S3: experimental laws of the normalized resistant area, $A_{res}/A_{n}$, and of the damage parameter, $D_{2}$, for variable slenderness of a cylindrical concrete specimen (uniaxial compression test), Figure S4: interpolating law of the average unloading–reloading slope for variable slenderness of a cylindrical concrete specimen (uniaxial compression test), Figure S5: failure mechanism of a cylindrical concrete specimen (uniaxial compression test), Figure S6: ratio of the transverse strain, $\varepsilon_{r}$, to the longitudinal strain, $\varepsilon_{l}$, with the transverse strain acquired both on the external surface and inside the resistant structure (concrete specimen in uniaxial compression), Figure S7: acquisition of the transverse strain, Figure S8: the values of $\varepsilon_{\vartheta }$, with the transverse strain acquired both on the external surface and inside the resistant structure (concrete specimen in uniaxial compression), Figure S9: stress/strain curves for variable slenderness of a cylindrical concrete specimen (uniaxial compression test), Table S1: description of the components of the LT mix.
