# Supplementary material for: Mechanical Properties of a 3D-Printed Wall Segment Made with an Earthen Mixture
Source: Materials (Basel). 2022 Jan 7;15(2):438. doi: 10.3390/ma15020438 (PMC8778666; doi:10.3390/ma15020438)
Supplement: Supplementary file 1 [file materials-15-00438-s001.zip › materials-1543585-supplementary.pdf]

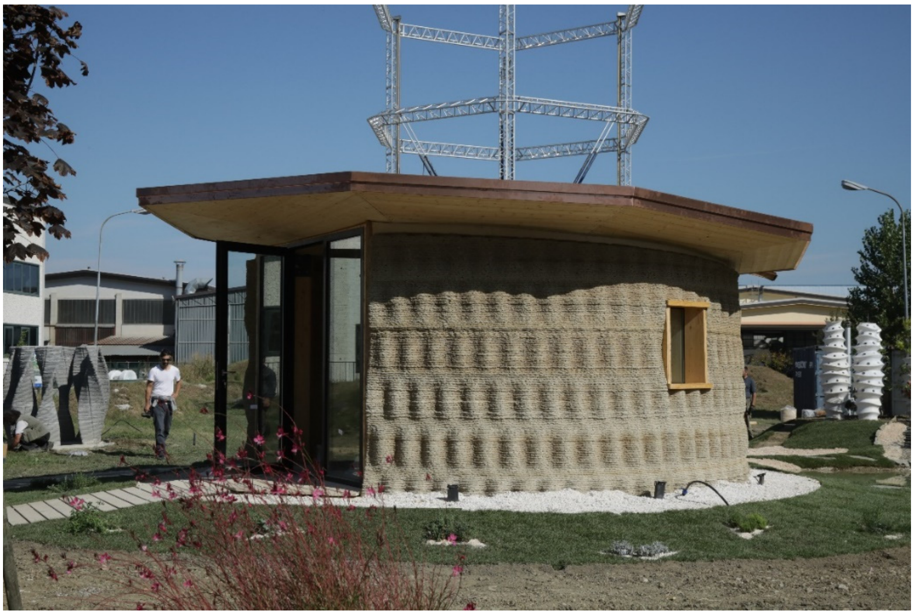

Figure S1. The Gaia House by WASP: first prototype of a 3D printed earthen building, with biodegradable materials (built in 2018) [source WASP].

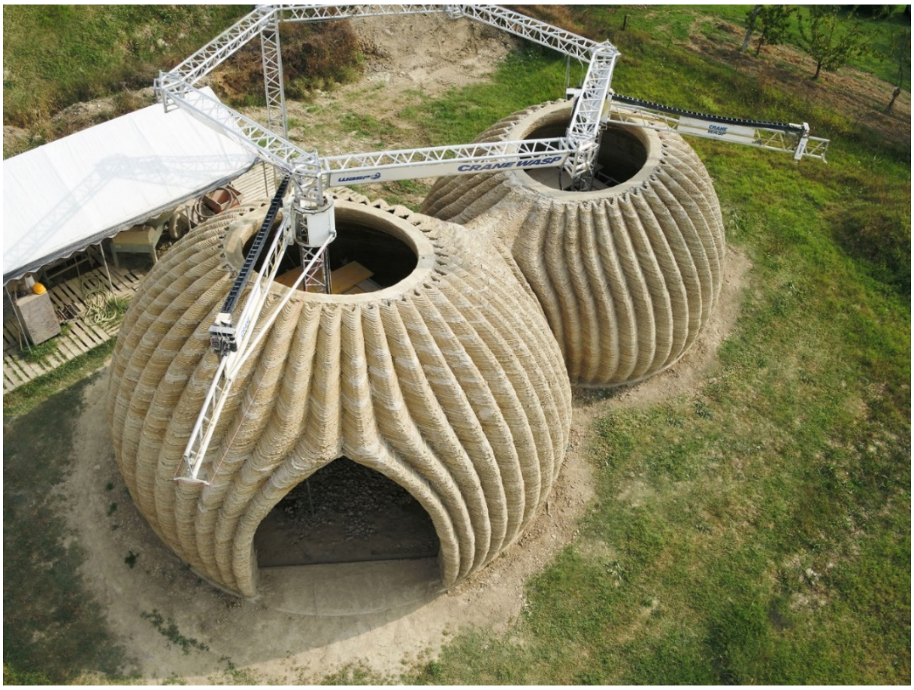

Figure S2. The Tecla House by WASP: 3D printing of the second prototype of an earthen building (completed in 2021) [source WASP].

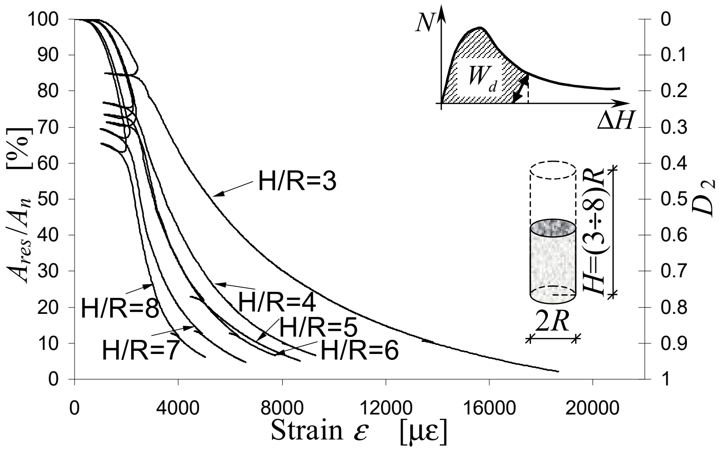

Figure S3. Experimental laws of the normalized resistant area, A_{res}/A_n, and of the damage parameter, D_2, for variable slenderness of a cylindrical concrete specimen (uniaxial compression test): A_{res} is the resistant area of the cross-section, A_n is the nominal area of the specimen before the test, D_2 is the damage parameter defined as the ratio of the dissipated energy in the generic step of the load test, W_d (the value of the area shown in the figure, included between the $N/\Delta H$ curve, the unloading path and the horizontal axis), to the total dissipated energy, $W_{d,t}$ ($D_2 = W_d/W_{d,t}$).

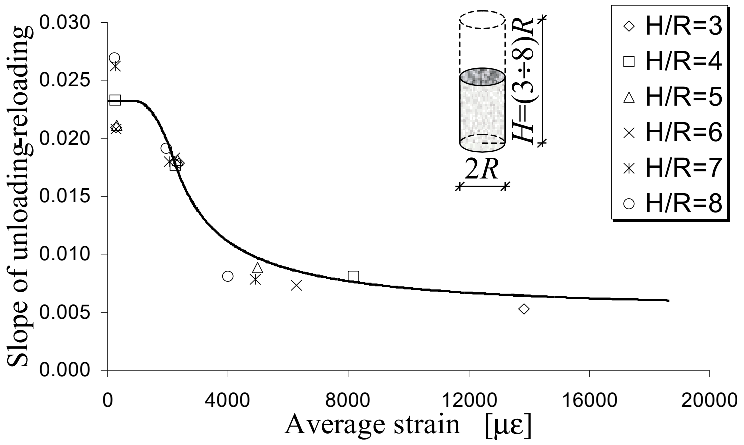

Figure S4. Interpolating law of the average unloading-reloading slope, for variable slenderness of a cylindrical concrete specimen (uniaxial compression test).

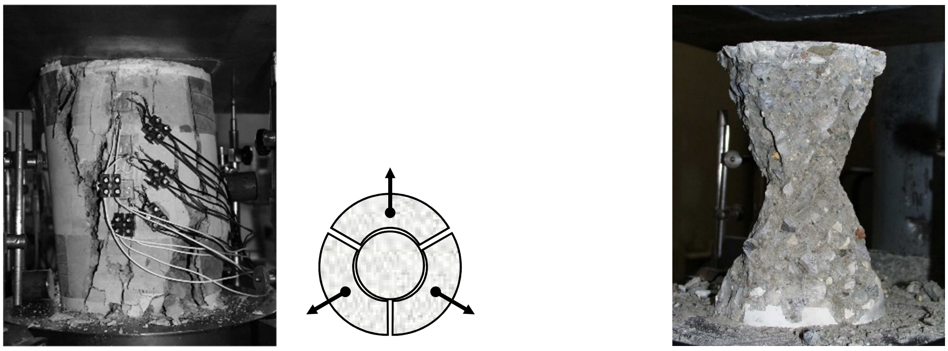

(**a**) (**b**)

Figure S5. Failure mechanism of a cylindrical concrete specimen (uniaxial compression test): (**a**) sub-vertical cracks on the external surface, originated by the expulsion of the external material due to the propagation of internal macro-cracks; (**b**) bi-conical resistant structure, emerging after removal of the damaged material.

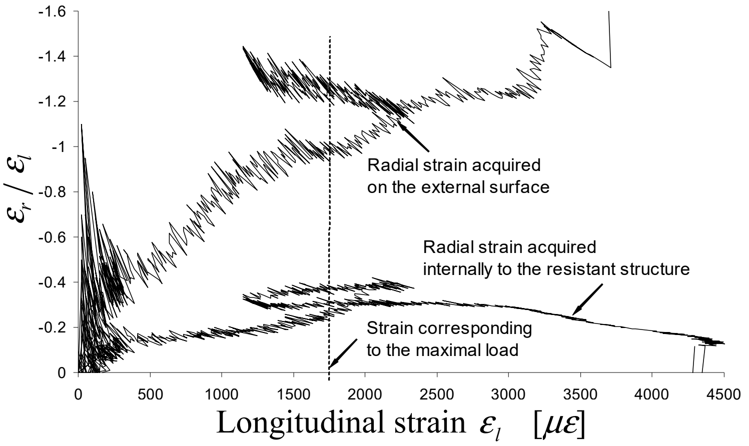

Figure S6. Ratio of the transverse strain, ε_r, to the longitudinal strain, ε_l, with the transverse strain acquired both on the external surface and inside the resistant structure (concrete specimen in uniaxial compression).

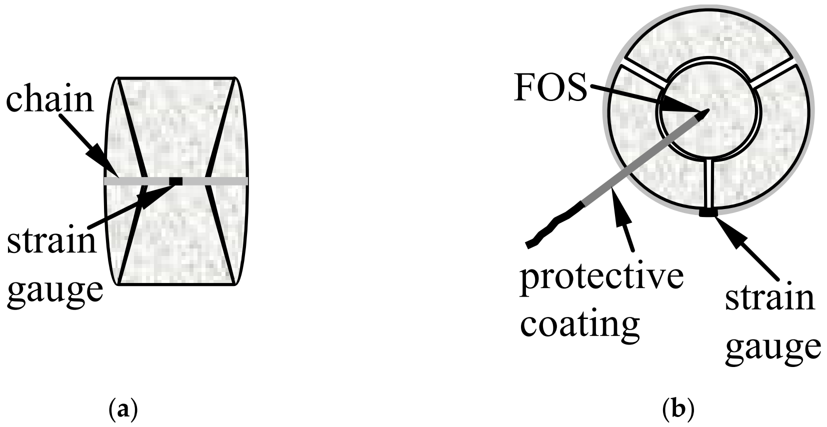

Figure S7. Acquisition of the transverse strain: (**a**) strain-gauge for acquisition of the circumferential strain on the external surface; (**b**) fiber optic sensor (FOS) for acquisition of the radial strain inside the resistant structure (cross-section view).

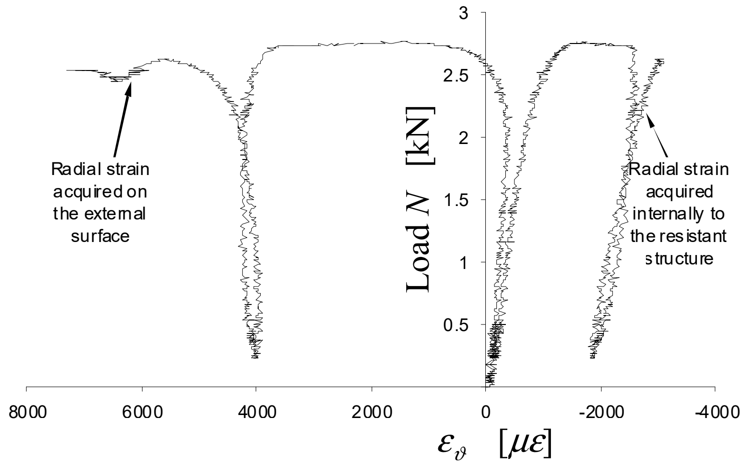

Figure S8. The values of ε_ϑ, with the transverse strain acquired both on the external surface and inside the resistant structure (concrete specimen in uniaxial compression).

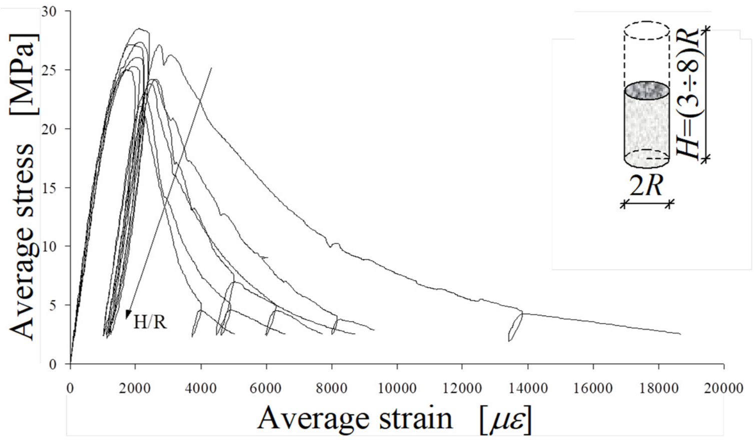

Figure S9. Stress/strain curves for variable slenderness of a cylindrical concrete specimen (uniaxial compression test).

Table S1. Description of the components of the LT mix.

Component	Description
Soil	It is the soil collected on site at the WASP headquarters, site of the 3D printing of the specimen. From the analysis of a soil sample, it emerged that the composition of the soil consists of 30% clay, 40% silt and 30% sand. Furthermore, the organic content of the soil (calculated according to ASTM D2974: 2000 directives) is 3.7%. According to the Highway Research Board (HRB)/UNI EN ISO 14688-1:2018 soil classification, this composition corresponds to a soil of class A-4, that is, a calcareous loam. Its optimum dry density is 1815 kg/m^3 (EN13286-2: 2005).
Lime-based binder	It is a high performance fiber-reinforced powder stabilizer with hydraulic action for the treatment and consolidation of soils and recycled or first-use aggregates. Its composition includes hydraulic lime for 25–50% and hydrated lime for 20–25%. The presence of specially selected mineral additions with pozzolanic activity (not deriving from the use of cement) and having binding properties (> 22% by weight) significantly increases the durability of the hardened mixture as well as the resistance to leaching of the stabilized material. In addition, the polypropylene fibers present in the product (dosage ≥ 0.1%), although with an aspect ratio greater than 600, are easily dispersed in the mixture and improve the final mechanical performance of the treated soil.
Hydraulic lime	Both the hydraulic lime contained in the lime-based binder and the one added separately have the function of allowing the carbonation to begin when the mixtures are still in their fresh state, which reduces setting time. In fact, since the aerial lime hardens in contact with the CO_2 contained in the air, in the absence of hydraulic lime the carbonation would begin only after the drying of the mixtures. Having a carbonation that begins immediately after mixing with water is instead mandatory to meet buildability. To this end, each printed layer must be strong enough to withstand the weight of subsequent layers before hardening and achieving some degree of structural integrity. When the mixtures dry, the hydraulic lime exhausts its function and the carbonation continues thanks to the fraction of aerial lime.
Silica sand	The (wet) silica sand added to that already contained in the soil has fluvial origin. Its grain size is between 0.00 and 0.60 mm.
